# Precision imaging of cardiac function and scar size in acute and chronic porcine myocardial infarction using ultrahigh-field MRI

**DOI:** 10.1038/s43856-024-00559-y

**Published:** 2024-07-18

**Authors:** David Lohr, Alena Kollmann, Maya Bille, Maxim Terekhov, Ibrahim Elabyad, Michael Hock, Steffen Baltes, Theresa Reiter, Florian Schnitter, Wolfgang Rudolf Bauer, Ulrich Hofmann, Laura Maria Schreiber

**Affiliations:** 1 Comprehensive Heart Failure Center (CHFC), Chair of Molecular and Cellular Imaging, University Hospital Wuerzburg, Wuerzburg, Germany; 2 Department of Internal Medicine I, University Hospital Wuerzburg, Wuerzburg, Germany

**Keywords:** Heart failure, Myocardial infarction, Magnetic resonance imaging

## Abstract

7 T cardiac magnetic resonance imaging (MRI) studies may enable higher precision in clinical metrics like cardiac function, ventricular mass, and more. Higher precision may allow early detection of functional impairment and early evaluation of treatment responses in clinical practice and pre-clinical studies. Methods: Seven female German Landrace pigs were scanned prior to and at three time points (3–4 days, 7–10 days, and ~60 days) post myocardial infarction using a whole body 7 T system and three radiofrequency (RF) coils developed and built in-house to accompany animal growth. Results: The combination of dedicated RF hardware and 7 T MRI enables a longitudinal study in a pig model of acute and chronic infarction, providing consistent blood tissue contrast and high signal-to-noise ratio (SNR) in measurements of cardiac function, as well as low coefficients of variation (CoV) for ejection fraction (CoV_intra-observer_: 2%, CoV_inter-observer_: 3.8%) and infarct size (CoV_intra-observer_: 8.4%, CoV_inter-observer_: 3.8%), despite drastic animal growth. Conclusions: Best results are achieved via manual segmentation. We define state-of-the-art procedures for large animal studies at 7 T.

## Plain language summary

In magnetic resonance imaging (MRI), scanners use magnets to generate detailed images of structures in the body, such as the heart. Stronger magnets can produce stronger magnetic fields, which can be leveraged for better image quality and developing new methods for disease diagnosis. In clinical practice, such systems using strong magnets are not yet used for imaging of the heart and some safety aspects remain challenging. We apply such an imaging approach in pigs, in which heart structure and function are similar to humans. We focus on the most important clinical imaging aspects following a heart attack, namely heart function and scar detection. We demonstrate that the high magnetic strength system enabled consistent image quality and accuracy. These findings may help to guide future developments in MRI of the heart, for example in patients who have had a heart attack.

## Abstract (web summary)

Lohr et al. demonstrate that 7 T cardiac MRI in conjunction with dedicated radiofrequency hardware enables high precision imaging of cardiac function and scar size in a large animal model of acute and chronic myocardial infarction. High precision is achieved based on consistent blood tissue contrast and SNR in measurements of cardiac function.

## Introduction

Cardiac magnetic resonance (CMR) imaging at 1.5 and 3 T is an accurate and highly reproducible technique for the assessment of cardiac function and ventricular mass in three dimensions^1^. Moreover, it provides unique parameters allowing tissue characterization which can be used e.g., for long term risk stratifications post myocardial infarction (MI)^2^. Still, improvements in standardization, faster scan times, contrast agent free scans, and tools for risk stratification regarding adverse remodeling and arrhythmic events remain highly desirable^3^.

For the assessment of cardiac function and ventricular mass, multiple 7 T studies have shown general feasibility and image quality at least comparable to that at clinical field strengths^4–8^. While technical and methodological challenges related to B_0_- and B_1_-nonuniformity remain, ultrahigh field (UHF, ≥7 T) magnetic resonance imaging (MRI) has demonstrated the potential to increase signal-to-noise ratio (SNR) and thus image resolution in CMR in humans^5,9^. These improvements may enable higher precision in cardiac function and left ventricular mass, providing means for early detection of functional impairment as well as early evaluation of treatment responses in clinical practice, but also in basic cardiovascular science or drug development studies. For such studies the increase in accuracy and reproducibility would enable smaller numbers of volunteers and patients to assess methodology or treatment efficacy.

Prior to clinical trials, which are required to evaluate safety and efficacy of new therapies, preclinical studies in large animals are needed^10,11^. In the context of ischemia-reperfusion, large animal models such as swine or dogs are selected, because anatomical features of the heart are more similar to humans than in rodents^12^. These models benefit from MRI technology on one hand^13,14^. On the other hand, they offer ideal testing conditions for method development and feasibility assessments in imaging regimes considered unacceptable (safety, duration) for humans. In addition, due to the high anatomical and physiological similarity, large animal models are also of wide interest in biomedical engineering in general^15–17^.

While large animal ischemia-reperfusion models have been used in preclinical cardiac MRI, these studies have been performed using clinical MRI systems at field strengths of 1.5 T^18–24^ and 3 T^23,25–27^. Radiofrequency (RF) coils in these systems are developed for human application and only provide a suboptimal fit in large animals due the difference in thorax dimension and shape, which results in limited SNR and thus, limited image quality and spatial as well as temporal resolution.

We hypothesized that a combination of dedicated RF hardware and UHF-MRI in large animal models will leverage the increase in SNR towards high precision quantification of CMR metrics like cardiac function, pushing accuracy and reproducibility. The primary aim of the study was the establishment of large animal 7 T cardiac MRI that enables longitudinal studies in a pig model of acute and chronic infarction. The secondary aim was to quantify image quality such as SNR and blood tissue contrast to demonstrate the feasibility of high-precision cardiac MRI in such studies.

We demonstrate that UHF MRI allows for high-precision longitudinal assessment of myocardial morphology, function, and scar development in a large animal model of acute and chronic MI. We further show the general feasibility of LGE imaging following MI using a whole-body 7 T MRI system. Combining dedicated RF coils, tailored MRI protocols, and a 7 T MRI system as key components, we present a new tool for high precision quantitative studies of cardiac function and infarct size.

## Methods

### General experimental setup for 7 T-based precision cardiac MRI in pigs

Our approach to address difficulties associated with 7 T in pigs is based on a set of three dedicated, shape-adapted, in-house developed RF coils (8 transmit and 16 receive channels, see Supplementary Fig. 1). The combination of these hardware developments, UHF CMR expertise^5,28^, and optimized MRI protocols, define state-of-the-art for translational studies, aiming to leverage benefits of UHF MRI for research in large animal models, interventions, drug trials, and methodology advancements towards human 7 T cardiac MRI. Schreiber et al. provide a detailed description of the infrastructure used and the hygiene concept applied here^29^.

All MRI measurements were performed using a whole-body 7 T MAGNETOM™ Terra system (Siemens Healthineers, Erlangen, Germany) with a partial 3rd order shim system^28^ and the three in-house built 8 Tx/16Rx cardiac transceiver arrays based on an antisymmetric mono-surface array design^30^ (Supplementary Fig. 2). The longitudinal setup of this study led to significant increases in animal weight from scan to scan and thus, variations in the thorax dimension and shape. The three transceiver arrays were built and optimized to accompany these variations (coil 1: 25–40 kg, coil 2: 40–60 kg, coil 3: ≥60 kg), aiming to maintain homogenous RF excitation, high SNR in images, and to enable adequate saturation and inversion pulses for CMR. Changes in the housing shape indicate how the thorax of the observed animals develops over time.

Cardiac gating was performed using an external acoustic triggering device (EasyACT, MRI Tools, Berlin, Germany). The vendor electrocardiography (ECG) was used as a control when the animal was still outside the bore. Anesthesia, breathing, and breath holds for imaging were controlled using a ventilator certified for MRI at 3 T (Julius, Dräger, Lübeck, Germany) and a portable bedside capnograph (N-85, Nellcor, Covidien, Mansfield, USA). Hydration and medication were delivered using Space® infusion pumps, shielded by a MRI safe (≤3 T) SpaceStation (Braun, Melsungen, Germany). Monitoring of animal body temperature (rectal) as well as oxygen saturation and heart rate was done using a fiber-optic sensor (Optocon AG, Dresden, Germany) and a pulse oximeter (8600FO, Nonin Medical Inc., Plymouth, USA), respectively. Contrast agent (Gadovist®) was applied using an injection system (MEDRAD® Spectris Solaris EP, Bayer AG, Leverkusen, Germany). A water-based heating mattress (Hico aquaterm 660, Hico Medical Systems, Cologne, Germany) was used, if the animal exhibited low body temperature prior to or throughout the MRI measurements.

### Animal experiments

Animals observed in this study were *n* = 8 female German Landrace pigs. All experiments were approved by local authorities (project 55.2.2 DMS 2532.2-1134-16, Government of lower Franconia, Germany). All operations and MR measurements were performed under anesthesia which was introduced intramuscularly using atropine (0.5 mg/kg), azaperone (8 mg/kg), and ketamine (15–20 mg/kg). Anesthesia was maintained intravenously using propofol (2–10 mg/kg/h) and fentanyl (0.01–0.03 mg/kg/h). Prior to MRI measurements, pigs were fasted for 16 h. Following in vivo measurements in MRI4, animals were euthanized using pentobarbital (150 mg/kg). Housing conditions were as described by ref. ^31^. Animals are indexed with letters A-H, easing attribution of images in this paper as well as in potential future publications relating to this manuscript.

### Study protocol

This work focuses on the feasibility of ultrahigh field CMR in a longitudinal large animal study, analyzing the consistency in image quality as well as the precision in derived CMR metrics. Supplementary Fig. 2 shows an illustration of the workflow throughout this study. Animals were given at least 10 days to rest and acclimate, following arrival. Afterwards, we placed two venous access ports into the jugular veins. After the first MRI (MRI1) measurement, which functioned as a baseline scan in the individual pig, MI was induced via 90 min balloon catheter occlusion of the proximal left anterior descending artery (LAD) with subsequent reperfusion. Follow-up MRIs (MRI2, MRI3, and MRI4) were performed 3–4, 10–14, and ~60 days post MI, respectively. Once in vivo measurements for MRI4 were done, animals were euthanized and a small set of images was acquired ex vivo *(*in situ*)*. After these measurements animals were removed from the scanner and hearts were excised in the operating theater.

### MRI protocol in vivo

Sequence parameters are listed in the Supplementary Material. All MRI measurements were performed under breath hold (exhaled, ventilator-induced) condition. Measurements started with a localizer for transversal, coronal, and sagittal slices at the approximate anatomical position of the heart. Based on this localizer we assessed whether the RF coil position was selected properly. Our RF coils are optimized to enable high SNR and a homogeneous B_1_ for the area of the heart. However, this optimization is based on certain assumptions such as thorax dimensions and coil position with respect to the heart. Suboptimal placement of the coil can therefore lead to suboptimal SNR or even destructive interference in the heart region. As an internal quality control mechanism, images gained from the localizer had to satisfy two conditions: (1) signal intensity maximum is centered on the heart and (2) minimal non-uniformity in signal intensity or destructive interference is observed within the heart region. If either of these conditions was not satisfied, the animal was removed from the bore, and the coil was repositioned with respect to the pig.

The localizer was also used to place a cuboid target region of interest (ROI) for B_0_-shimming around heart. A dedicated sequence was run with the sole purpose of measuring a B_0_-map, which is used to calculate and apply required shim currents. We used the vendor-supplied B_0_-mapping approach with the shortest echo time (TE) and echo spacing.

Cardiac planning was performed using a gradient echo (GRE) sequence in order to obtain short axis, long axis and 2-chamber views. Prior to further measurements, we ran short axis and long axis CINE measurements with 15°, 20°, and 25° flip angle values (calibrated by vendor-integrated procedure) to optimize the CINE image quality regarding blood-tissue contrast (BTC) and minimal flow-induced artifacts. This was essential, because the scanner integrated flip-angle calibration provided inconsistent contrast in 7 T CMR. All CINE acquisitions in this study are based on a T_1_-weighted spoiled GRE sequence. On average, this automatic calibration set the transmission reference voltage to $\overline{{MRI}1}$ = 342 V, $\overline{{MRI}2}$ = 334 V, $\overline{{MRI}3}$ = 340 V, and $\overline{{MRI}4}$ = 406 V. In cases where the contrast was not optimal for one of the 5° increments, an intermediate value, e.g., 18° was selected. All subsequent measurements applied this flip angle and a slice thickness of 6 mm. For the evaluation of cardiac function, we acquired CINE data for a short axis stack with 0.6 × 0.6 mm^2^ and 0.4 × 0.4 mm^2^ in-plane resolution. CINE data was also acquired for the long axis and 2-chamber view.

In order to enable signal inversion in late gadolinium enhancement (LGE) imaging, sufficiently high flip angles (180° pulse) are required. Experience from iterative RF power calibration with respect to requirements for magnetization inversion performed in training scans prior to this study was used to set reference voltages sufficiently high before contrast agent application. Depending on animal size and the coil used, the voltages were increased by 50–70% to enable the signal inversion required for LGE, resulting in average system transmission reference voltages of $\overline{{MRI}1}$ = 517 V, $\overline{{MRI}2}$ = 570 V, $\overline{{MRI}3}$ = 593 V, and $\overline{{MRI}4}$ = 600 V in this study.

Contrast agent (weight [kg]/10) was injected and flushed (4 ml/s) using 40 ml saline solution.

After contrast agent application we waited for ~8 min to run three inversion recovery sequences with inversion times (TI) of 350, 400, and 450 ms, to determine the optimal TI for LGE (Supplementary Fig. 3). With typical inversion times around 350 ms at 3 T for waiting periods of >8 min, we estimated these three inversion times to provide sufficient information to assess nulling of the myocardial signal. The higher inversion times were meant to consider increased T_1_ relaxation times at 7 T. Longer inversion times than 450 ms were only applied when myocardial nulling was not achieved, and the individual heart rate of the animal allowed. Typical lengths of RR intervals observed in our animals ranged from 658–1402 ms. LGE measurements were done for the short axis stack, the long axis, and the 2-chamber view. Both, magnitude and phase-sensitive (referred to as PSIR: phase sensitive inversion recovery) images were generated.

Final scans for the in vivo protocol were transversal, coronal, and sagittal gradient-echo sequences with the optimal flip angle and vendor B_1_ maps for corresponding image positions. This data was acquired to enable a more meaningful comparison of SNR throughout the study.

### MRI protocol in situ

Prior to euthanasia, we applied another 3 ml of contrast agent which was allowed to circulate for ~5 min. Cardiac arrest was induced outside the bore. The patient table position was not reset, allowing re-entry of the animal to the previous measurement position. The ex vivo protocol was kept short to enable later excision and histologic assessment of the heart. High-resolution LGE (0.4 × 0.4 mm^2^) data was acquired using slice positions of the in vivo short axis stack in order to verify our in vivo results in the absence of motion.

### TTC staining post mortem

After in situ measurements of LGE the animal was removed from the scanner bore and brought to the operating room. The heart was removed from the chest cavity and excess blood rinsed with isotonic saline solution. Hearts were stored in saline solution and transported to the laboratory, where all major vessels, both atria, and the right ventricle were surgically removed. Left ventricle (LV) tissue was then frozen at −80 °C for a duration of 45 min. Afterwards, slices with a thickness of 5 mm were prepared using a cutting machine (AMH250A, GGM Gastro International, Germany). A 1% tetrazolium chloride (TTC) solution was used for staining. Each slice was placed in the staining solution and incubated for 20 min at 38 °C using a shaker system (Multitron Standard, Infors GmbH, Germany) with 50–60 rotations per minute. Aiming to provide data that would be more comparable to the way MRI acquisitions work, each slice was photographed with line of sight from base to apex as well as apex to base post staining. These images are subsequently referred to as top and bottom views, respectively. Every image also contained a ruler aligned with the LV tissue to enable estimation of the infarct area.

### Data analysis

Cine data (end-systole and end-diastole) was manually segmented using the CMR software Medis Suite™ (Medis, Leiden, Netherlands). Myocardial contours were exported from Medis and converted to png-files using Python^32^.

All data analysis with respect to BTC and myocardial SNR was done using in-house developed Matlab (Mathworks, Natick, USA) code and these png-contour-files. BTC was calculated as BTC = S_b_/S_myo_, where S_myo_ is the mean signal intensity of all pixels within the contours of the left ventricular myocardium, and S_b_ denotes the mean signal intensity of the whole blood pool. Since the blood pool segmentation also contained signal from the papillary muscle, we applied a filter to the blood pool which removed all pixels where the signal was within one standard deviation of the mean myocardial signal (Supplementary Fig. 4). Blood pool data was filtered this way slice by slice. Visual assessment of this process demonstrated good agreement between papillary muscle and voxels removed from the blood pool signal (Supplementary Fig. 4).

Myocardial SNR (SNR_myo_) was calculated as SNR_myo _= *S*_myo_/*σ*, where *S*_myo_, denotes the mean signal intensity of the left ventricle, and σ the standard deviation of noise approximated within a non-signal-producing area of the images (50 × 50 pixel). As proposed by ref. ^33^. the standard deviation of noise was approximated as half of the root mean square value of all pixels (L) within that non-signal-producing area divided by twice the number of receivers (*n* = 16) used.

1 $$\sigma =\sqrt{\frac{{\sum }_{i=1}^{L}{\left({pixel\; value}\right)}_{i}^{2}}{2{Ln}}}$$

Myocardial SNR on a pixel-by-pixel basis was calculated as SNR_pixel _= *S*_pixel_/*σ*, where S_pixel_ is the signal intensity of that pixel and *σ* is the standard deviation of noise as described above.

The geometric distance between the measured tissue and the coil and its individual elements directly influences the achievable SNR. Due to the use of a coil array in combination with adaptive image reconstruction of the MRI system where receive coils with more signal are weighted higher in image combination, assessing the influence of distance on SNR numerically, becomes complex. We therefore only provide a *distance weight* (denoted as *W*_d_) in addition to raw SNR values. In order to calculate this weight, we measured the shortest distance from the center of the LV to the coil using mid-cavity GRE images acquired with very short echo time (1.1 ms). In combination with logarithmic scaling of signal intensity these images enabled visualization of the coil borders and the measurement of the geometric distance (Supplementary Fig. 5). Respective values were then corrected for the offset in angulation with respect to the true transversal orientation that was required for the short axis acquisition. The corrected values were then normalized to the maximum distance, creating the distance weight *W*_d_. The closer the value gets to 1, the higher the influence of coil-tissue distance on SNR.

In addition, there is no body coil available at 7 T that would enable RF excitation of the whole animal. Instead, excitation is achieved via multiple local transmit elements in the RF coil, which increases the influence of the distance between transmit elements and our target tissue on SNR compared to clinical systems. In order to distinguish between SNR loss caused by the receive and the transmit pathway of the MRI system, we measured flip angle values in the anterior wall of the heart. For this purpose a stack of B_1_ maps was measured with the vendor-integrated pre-saturated GRE pulse sequence^34^ in transversal orientation in each session. Data quality of the B_1_ maps was limited, because the sequence did not provide means for cardiac gating in reasonable scan times. This led to areas without information in vendor reconstructed B_1_ maps. Since areas with missing information varied from slice to slice and B_1_ variations are typically smooth in the absence of interference patterns, we performed a maximum intensity projection for the five central slices of the heart to compensate for signal dropouts in the vendor reconstruction of these maps. We then placed a circular ROI on the intersection of left and right ventricle in the anterior wall of the maximum intensity projection to provide an estimate of flip angle (Supplementary Fig. 6). Average flip angles (FA) in each individual scan were subsequently normalized to the maximum flip angle of all scans. SNR of the receive pathway of the MRI system was then estimated as:

2 $${{SNR}}_{{receive}}=\frac{{{SNR}}_{{myo}}}{{\sin }({FA}_{{norm}})}$$

where SNR_myo_ is the myocardial SNR as described above, and FA_norm_ denotes the normalized flip angle in the anterior wall.

To assess intra-observer variability in segmentation and resulting ejection fraction, all data was segmented twice (AK). In addition, all data was segmented by another observer (DL) to determine inter-observer variability. Neither observer had access to segmentations from the other observer and was thus performed blinded. As part of the inter-observer variability assessment, we checked if there were differences in the cardiac phases selected as end-systole and end-diastole. Matching end-systole and end-diastole to identical phases for our two observers, we determined the inter-observer variability solely due to segmentation. We refer to this process as “phase harmonization” of CINE data analysis. The coefficient of variation for inter- and intra-observer variability was calculated as the single standard deviation of the difference divided by the mean of the two measurements.

LGE data (both magnitude and PSIR images) was manually segmented using the Medis. Myocardial contours were drawn for endocardium and epicardium to provide ventricular volume and mass. Both hypo- and hyperintense areas in the infarct area were manually labeled. Infarct sizes in % of total volume and in grams were automatically calculated by Medis. In order to assess intra-observer variability in segmentation and infarct size, all data was segmented twice (AK). For inter-observer variability data was also segmented by another observer (DL). In addition infarct areas were determined using the following semi-automatic approaches: full width at half maximum (FWHM) as well as 3SD, 5SD, and 7SD, where SD stands for standard deviation. All these methods also require segmentation of endocardial and epicardial contours. We used contours drawn by AK for the application of all semi-automatic approaches. For the FWHM method an additional ROI is drawn in the brightest section of the infarct. All voxels within the myocardial borders, where the signal intensity is above 50% above of the maximum signal intensity within the drawn ROI are labeled as infarct. For the standard deviation methods, an additional ROI is drawn in the healthy myocardium rather than in the infarcted tissue. All voxels within the myocardial borders, where the signal intensity is outside 3, 5, and 7 standard deviations of the signal intensity within the healthy myocardium is labeled as infarcted tissue. Infarct sizes in % denote the fraction of infarcted myocardium relative to the total LV mass. Labeling of contours and myocardial ROIs for semi-automatic approaches was performed twice to provide intra-observer variability (AK).

For infarct size quantification based on the images of TTC stained myocardial slices, we applied the open-source software tool ImageJ (version 1.53k). Analogous to segmentation in Medis MR Suite, endocardial end epicardial borders of the LV were delineated as well as the infarct area, which had remained white, while viable myocardial tissue had turned red post TTC staining. Infarct quantification in % of total LV volume was performed in this fashion for both top and bottom views as well as the mean of both views.

In order to assess whether discontinuities between slices due to sawing occurred, we subtracted the infarct size from every top view from the bottom view of the slice. Respective difference values were then normalized to the total infarct size to determine the impact of discontinuity on a slice-by-slice basis.

The impact of the line of sight (top versus bottom view) on infarct size was assessed in a similar way. We first calculated respective differences in each slice (top view minus bottom view of the same slice) and then normalized the values to the total infarct size again.

Tissue preparation for TTC staining led to changes in LV dimensions (different tissue and contraction states) compared to in vivo and in situ images. In addition to infarct sizes reported as a fraction of the total myocardial mass, we therefore also analyzed infarct sizes based on weight in both LGE and TTC data to assess whether this improved comparability between the applied methods. While Medis also provides the infarct size in g directly, this metric had to be calculated for TTC data. We used the ruler contained in every image to estimate the pixel dimension and subsequently the infarct size in g, assuming a tissue density of 1.05 g/cm^3^_._

### Statistics

All statistical tests were done using Origin 9 (OriginLab) and MATLAB. Data such as ejection fraction (EF), BTC, and infarct size was analyzed for normal distribution using a Shapiro-Wilk test with *p* < 0.05. Subsequent tests for significant differences (*p* < 0.05) in our EF data were performed using ANOVA with Tukey post-hoc test. EF values (MRI1-MRI4) derived in the context of inter- and intra-observer were tested using a paired *t*-test with *p* < 0.05. Statistical tests for blood tissue contrast at MRI1, MRI2, MRI3, and MRI4 were done using a paired t-tests with *p* < 0.05 (diastole versus systole). Correlation analysis for ejection fraction (intra- and inter-observer variability) and the infarct size (intra- and –inter-observer variability) was done using the Pearson correlation coefficient and the coefficient of variation (CoV). For LGE we also determined the intraclass correlation coefficient (ICC; using a two-way random effect model, absolute agreement, and average measurement) in order to improve comparison to literature reports. Infarct sizes in % and in g determined manually as well as semi-automatic based on PSIR images were compared using a paired *t*-test with *p* < 0.05 (always against manual segmentation of Observer 1). CoVs and ICCs were determined for intra-observer variability of semi-automatic approaches. Infarct sizes (in % and g) measured in vivo (MRI4), ex vivo (MRI4) and based on TTC data were tested for similarity using a paired t-test with *p* < 0.05. Correlation analysis was done using the Pearson correlation coefficient. Analysis with respect to TTC data included infarct sizes determined based on the top view, the bottom view, and the mean of both views. Bonferoni correction was applied for multiple testing using paired t-tests, where applicable. Boxes in boxplots depict 25th to 75th percentiles, while medians are marked as horizontal line. Whiskers extend to the most extreme data points and outliers are indicated by red pluses.

### Sample size calculation

In order to show the importance of precision for pre-clinical and clinical studies we demonstrate its impact on sample size requirements comparable to ref. ^35^. Sample sizes (n) required to show a clinically meaningful change in EF with a power of 90% and an α error of 0.05 were calculated as:

3 $$n=f\left(\alpha ,P\right)* {\sigma }^{2}* \frac{2}{{\delta }^{2}}$$

where $f$ denotes a factor that depends on the significance level α and the study power *P* (for α = 0.05 and *P* = 0.90, $f$= 10.5), *σ* is the inter-observer pooled standard deviation and *δ* is the desired difference to be detected.

### Reporting summary

Further information on research design is available in the Nature Portfolio Reporting Summary linked to this article.

## Results

In order to ease data comparison with this study and future publications animals were labeled with letters (A-H). All animals except one finished the complete study protocol. Animal (D) died during infarct induction. Therefore, MRI data for animal D was only available at baseline and thus, omitted from this study. MRI was performed at four time points regarding stages of MI and will be subsequently be referred to as MRI1 (prior to MI), MRI2 (acute, 4 ± 1 days post MI), MRI3 (sub-acute, 4 ± 1 days post MI), and MRI4 (chronic, 58 ± 2 days post MI).

### Image quality in measurements of cardiac function

**Fig. 1:**
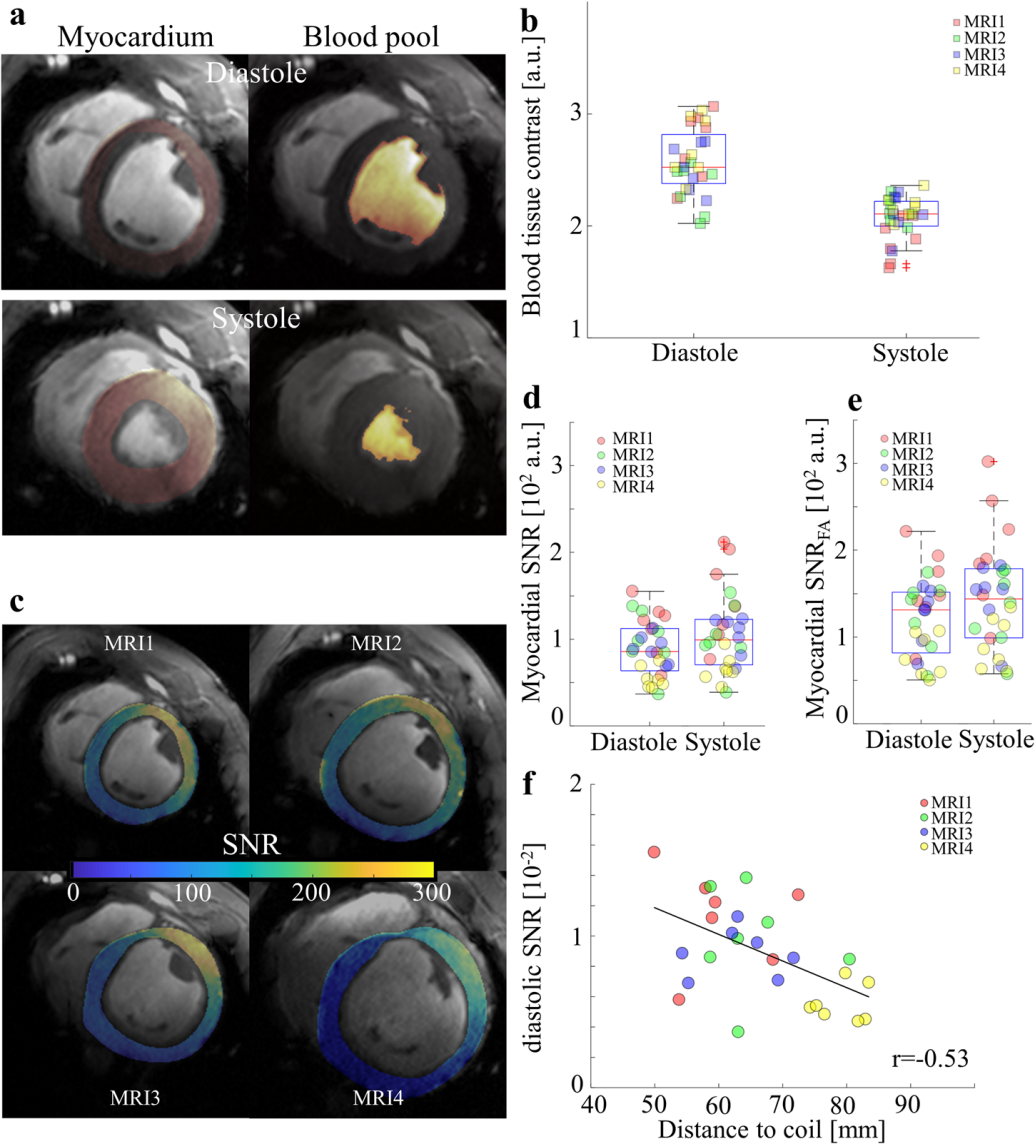
Blood tissue contrast and SNR in 7 T CINE MRI of animals (healthy and post MI) within a weight range of 30–82 kg. **a** Representative segmentation for a basal slice in systole. The area with red overlay corresponds to the myocardium, while the yellow area corresponds to the blood pool. The blood pool segmentation includes papillary muscle and cannot directly be used to get an average blood pool signal. We apply a filter that removes all voxels within a signal intensity of one standard deviation of the mean myocardial signal (Supplementary Fig. 4). The displayed blood pool contours are post-application of the filter. Papillary muscles are removed from the segmentation. **b** Blood tissue contrast for all measurements for diastole and systole. **c** Myocardial SNR maps (animal) for MRI1 (baseline, weight range: 30-45 kg), MRI2 (acute, 4 ± 1 days post MI, weight range: 35–50 kg), MRI3 (sub-acute, 12 ± 1 days post MI, weight range: 40-53 kg), and MRI4 (chronic, 58 ± 2 days post MI, weight range: 70–82 kg). Wall thinning in the anterolateral wall post MI can be seen particularly well for MRI3 and MRI4. **d** Raw, unmodified, myocardial SNR for diastole and systole. **e** Myocardial SNR after flip angle normalization based on measured B_1_ maps for diastole and systole. **f** Diastolic SNR plotted against the shortest distance measured from the LV center to the coil housing. This distance was measured using gradient echo images with very short echo time (1.1 ms). The expected negative correlation (Pearson, *n* = 28) is observed. Color-coding in (**b**), and (**d**–**f**) corresponds to measurement time points prior to and post MI. Boxes in boxplots depict 25th to 75th percentiles, while medians are marked as horizontal line. Whiskers extend to the most extreme data points and outliers are indicated by red pluses. Sample size in all plots is *n* = 28. LV left ventricle, MI myocardial infarction, MRI magnetic resonance imaging, SNR signal-to-noise ratio.

Flip angle values in each scan were visually optimized regarding BTC and SNR as described in the method section. Average flip angle values set for CINE and LGE acquisitions were 18 ± 2°, 17 ± 2°, 19 ± 3°, and 22 ± 5° in MRI1, MRI2, MRI3, and MRI4, respectively. To quantify BTC quantitatively we used myocardial and blood pool contours exported from Medis Suite™ software. Examples for myocardial contours are displayed in Fig. 1a. Average BTC values at the four measurement time points in diastole and systole for MRI1-MRI4 are listed in Table 1. Mean BTC values in end-diastole and systole throughout the study were 2.6 ± 0.3 and 2.1 ± 0.2, respectively (Fig. 1b). BTC in systole (range: 1.6–2.3) was significantly lower than in diastole (2.0–3.1) at most measurement points (*p*_MRI1_ = 4.1 × 10^−5^, *p*_MRI2_ = 5.3 × 10^−2^, *p*_MRI3_ = 2.2 × 10^−3^, *p*_MRI4_ = 4.3 × 10^−4^), while BTC within the same cardiac phase was rather consistent between MRI1 to MRI4, despite the large variation in animal weight and thorax size. BTC values for individual scans are listed in Supplementary Table 1.

**Table 1:** Parameters of image quality in the left ventricle derived from CINE MRI throughout the study

Metric	MRI1, baseline (Prior to MI)	MRI2, acute (4 ± 1 days pMI)	MRI3, sub-acute (12 ± 1 days pMI)	MRI4, chronic (58 ± 2 days pMI)
dBTC	2.7 ± 0.3	2.3 ± 0.2	2.5 ± 0.2	2.7 ± 0.3
sBTC	1.9 ± 0.2	2.1 ± 0.1	2.1 ± 0.2	2.2 ± 0.1
dSNR	113 ± 34	98 ± 34	89 ± 16	56 ± 12
sSNR	147 ± 51	102 ± 37	104 ± 22	66 ± 16
FA [°]	50.6 ± 5.8	48.9 ± 2.9	44.2 ± 5.4	43.7 ± 7.0
dSNR_B_1,norm_	158 ± 51	126 ± 42	126 ± 32	68 ± 18
sSNR_B_1,norm_	229 ± 76	146 ± 49	161 ± 39	89 ± 24
distance C-LV [mm]	58 ± 6	63 ± 5	65 ± 9	76 ± 5
W_d_	0.69 ± 0.07	0.76 ± 0.06	0.78 ± 0.11	0.91 ± 0.06

Mean values ± a single standard deviation.

The closer the W_d_ value gets to 1, the higher the influence of coil-tissue distance on SNR.

*MI* myocardial infarction, *pMI* post myocardial infarction, *dBTC* diastolic blood tissue contrast, *sBTC* systolic blood tissue contrast, *dSNR* diastolic SNR, *sSNR* systolic SNR, *dSNR_B*_*1*_*,*_*norm*_ diastolic SNR normalized to flip angle, *sSNR_B*_*1*_*,*_*norm*_ systolic SNR normalized to flip angle, *FA* average flip angle in the anterior wall derived from transversal B_1_ maps, *distance C-LV* shortest distance from coil housing to LV center, *W*_*d*_ distance weight (distance C-LV normalized to the maximum distance).

Myocardial SNR (whole short axis stack) reduced gradually with increasing animal weight (*r* = −0.64, Fig. 1f and Table 1). In diastole, raw myocardial SNR in MRI1, MRI2, and MRI3 was 2.0, 1.8, and 1.6 times higher than in MRI4. In systole, myocardial SNR was 2.2, 1.6, and 1.6 times higher, respectively. With increasing animal size, the filling factor of the coil typically decreases, which leads to lower receive sensitivity overall. In order to assess the receive efficiency of the coil we determined SNR values normalized to the sine of flip angle values in the anterior wall. Normalized SNR values (see Fig. 1e) display higher variance compared to raw SNR values (Fig. 1d). Increasing animal size also leads to higher distances from LV center to the coil, which effects both receive and transmit efficiency. On average, the closest distance from the coil to the center of the LV in MRI1, MRI2, and MRI3 was 58 ± 6, 63 ± 5, and 65 ± 9, which is 24%, 17%, and 14% less than in MRI4, respectively. SNR as a function of distance is plotted in Fig. 1f, depicting a correlation (*r* = −0.53) between observed myocardial SNR and the distance between the heart and the RF coil. The highest influence of coil-tissue distance was found for MRI4, as expected. Notably, due to the thorax size and shape, and particularly in younger animals, the shortest distance from the coil to the LV center was usually not from anterior to posterior but towards the flanks of the animal. SNR values for individual scans are listed in Supplementary Table 1.

Excellent image quality and high SNR allowed for a clear definition of the boundaries between myocardial tissue and the blood pool in CINE images. It also enabled tracking of small or thin anatomical structures such as coronary arteries and valves.

**Fig. 2:**
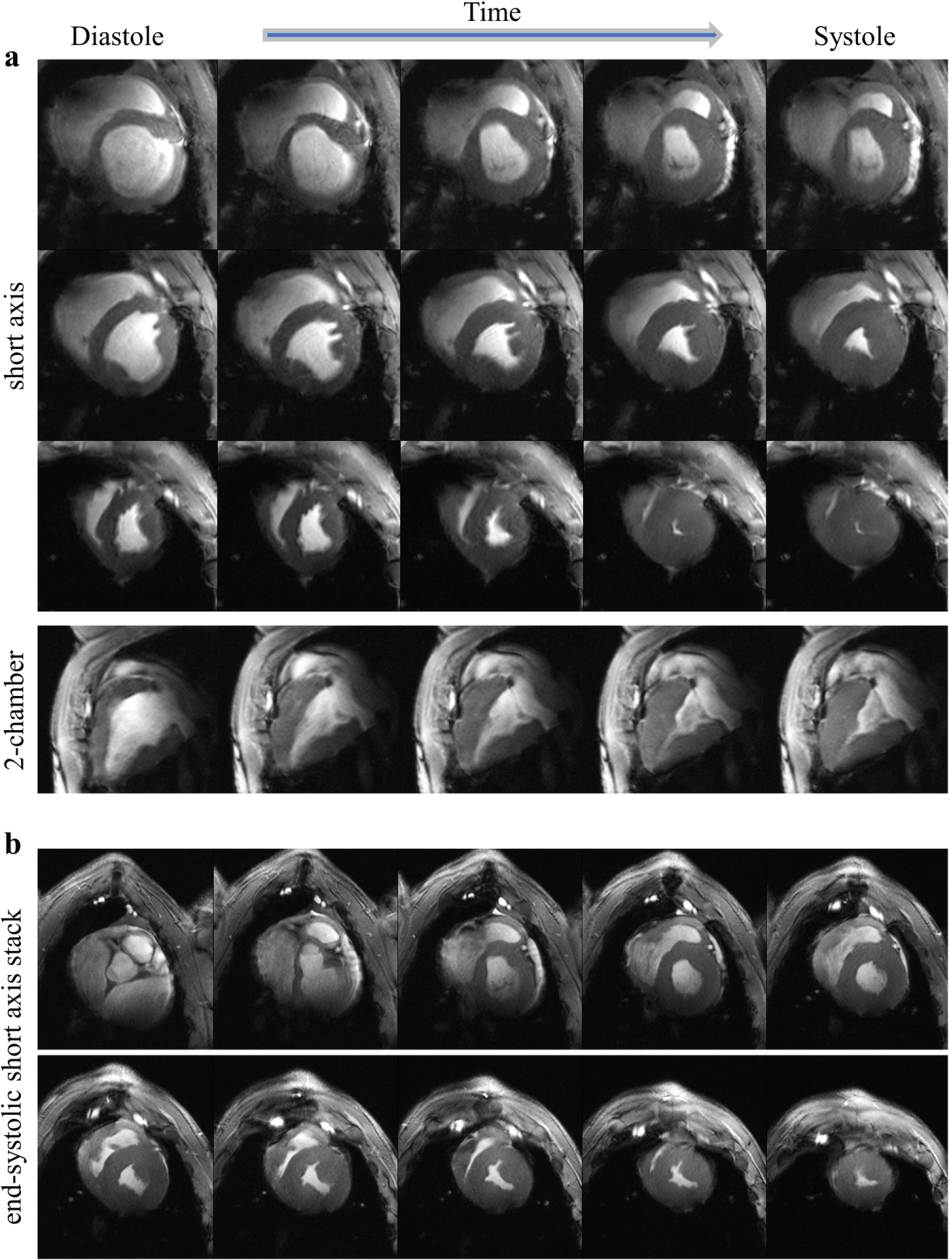
UHF cardiac MRI provides data on cardiac function with higher resolution than is feasible in clinical routine. Representative high resolution (0.4 × 0.4 mm^2^ in-plane) cine images of a baseline (healthy) MRI (animal F). To illustrate image quality over the cardiac cycle, five equidistant time points of a RR-interval are displayed. Images were acquired with coil1. **a** Short axis view for a basal, a mid-cavity, and an apical slice as well as a 2-chamber view. **b** End-systolic short axis stack. MRI magnetic resonance imaging, UHF ultra-high field.

**Fig. 3:**
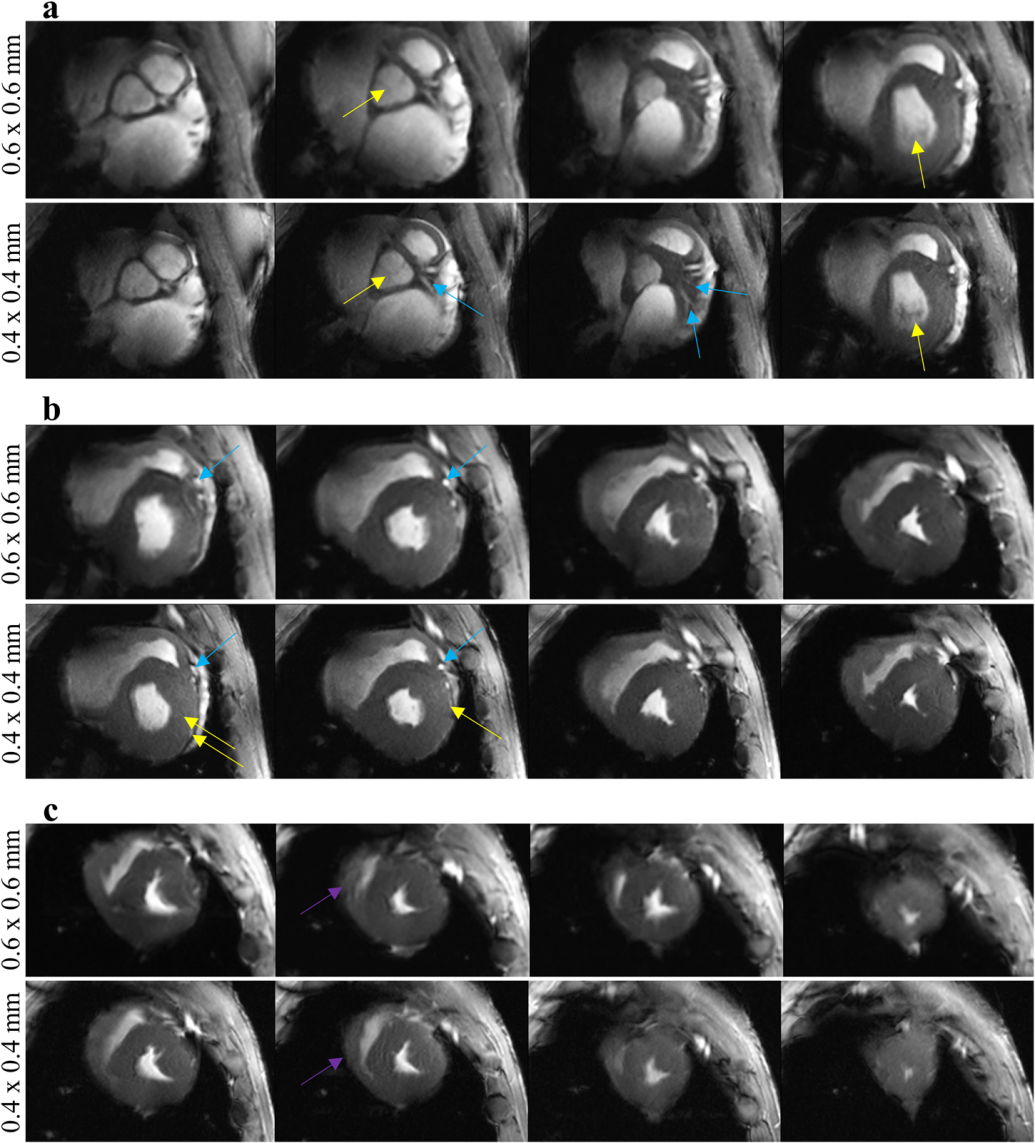
UHF cardiac MRI in a pig before infarct induction enables the visualization of fine structures currently not assessed in clinical routine and improves delineation of tissue boundaries. Comparison of moderate (0.6 × 0.6 mm^2^) and high spatial in-plane resolution (0.4 × 0.4 mm^2^), systolic, short axis CINE stack (animal F). Minor artifacts are visible in the right ventricle, in the left ventricle near blood-myocardium interfaces (dark rim artifact), and at lung-tissue boundaries. Arrows (yellow: valves, blue: vasculature, purple: RV) point towards examples for improved visibility of fine anatomical structures due to the higher resolution. **a** Basal segments in both resolutions. The increased resolution enables the identification branching coronary arteries. **b** mid-cavity segments in both resolutions. The increased resolution led to better delineation of coronary arteries and myocardial boundaries and indications of crypt-like features in the myocardium. **c** apical segments in both resolutions. The increased resolution led to better delineation of the RV. MRI magnetic resonance imaging, RV right ventricle, UHF ultra-high field.

Figure 2 shows that despite high spatial resolution (0.4 × 0.4 mm^2^) CINE images are free from motion induced blurring and maintain good temporal (30 cardiac phases per RR interval) resolution. At higher spatial resolution fine, anatomical details become apparent (Fig. 3). In general, the higher resolution improved clarity of blood-tissue-boundaries in both the left and right ventricle, although we focused on the LV in this study.

**Fig. 4:**
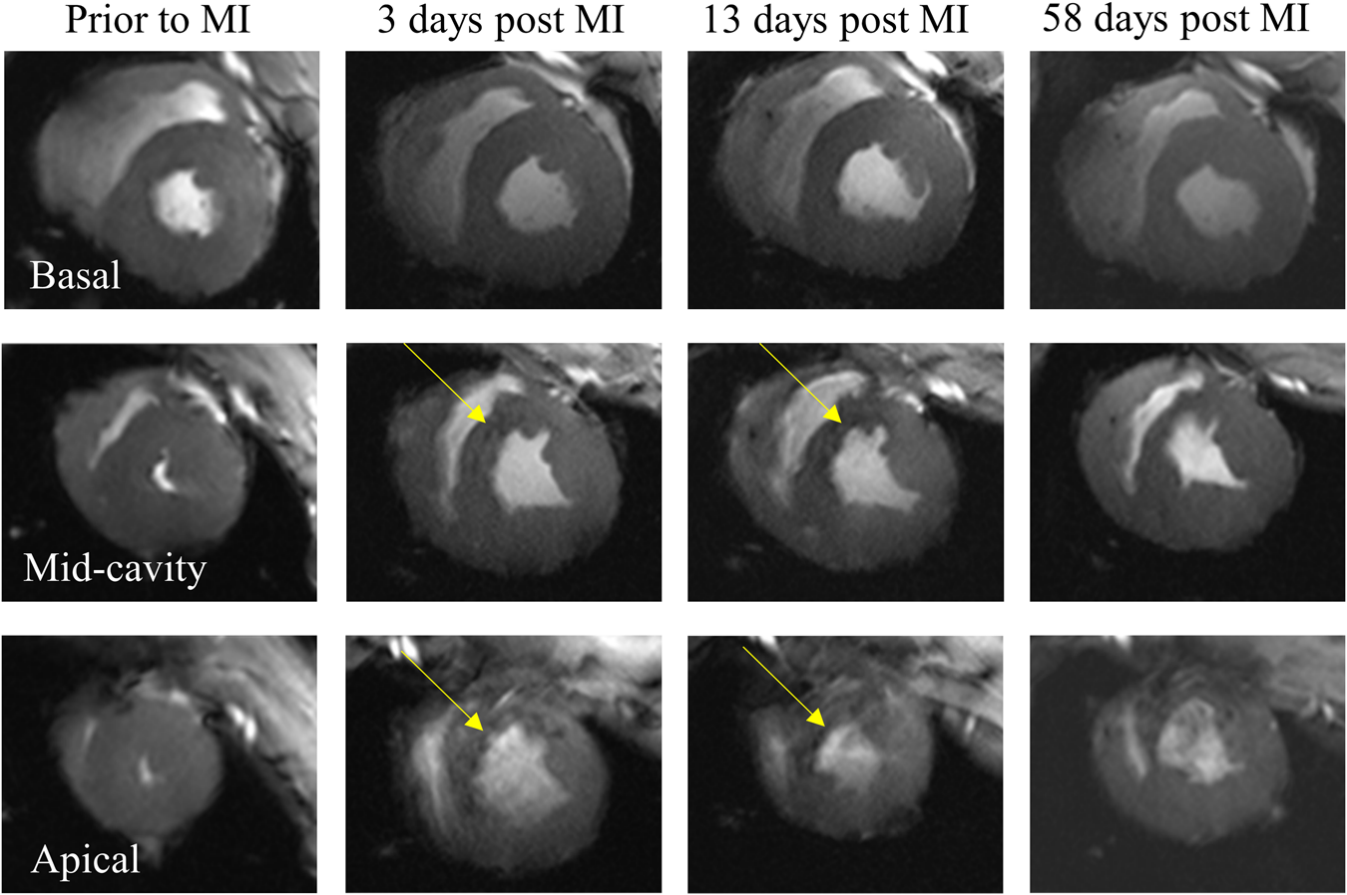
Susceptibility effects in CINE acquisitions at 7 T (animal H). Representative basal, mid-cavity, and apical CINE images of the same animal prior to and at three time points after myocardial infarction. Arrows indicate areas with susceptibility induced signal loss in mid-cavity and apical slices in the acute and sub-acute phase post MI, which are most likely caused by hemorrhage and structural changes in the myocardium after myocardial infarction. Susceptibility induced signal loss was significantly less severe in chronic stages of MI. Images in the chronic phase show severe wall thinning in the infarct region. MI myocardial infarction.

Myocardial wall motion and the integrity of contractile function were first visually assessed using the CINE data, which also showed significant wall thinning post-MI (see Fig. 4). As expected, LV contraction in mid-cavity and apical slices, in particular, was severely reduced after MI. Wall motion abnormality in these slices is illustrated in Fig. 4 and Supplementary Video 1. In CINE images of slices that exhibited wall motion abnormality, we also observed susceptibility induced signal loss in the infarct region (Fig. 4, yellow arrows). Visually, these areas of hypointensity correlated well with signal enhancement in LGE images, indicating that CINE images acquired with a TE of 2.9 ms using a GRE sequence (Fig. 4) are sensitive to differences between healthy myocardium and the infarct area.

### Analysis of cardiac function

**Fig. 5:**
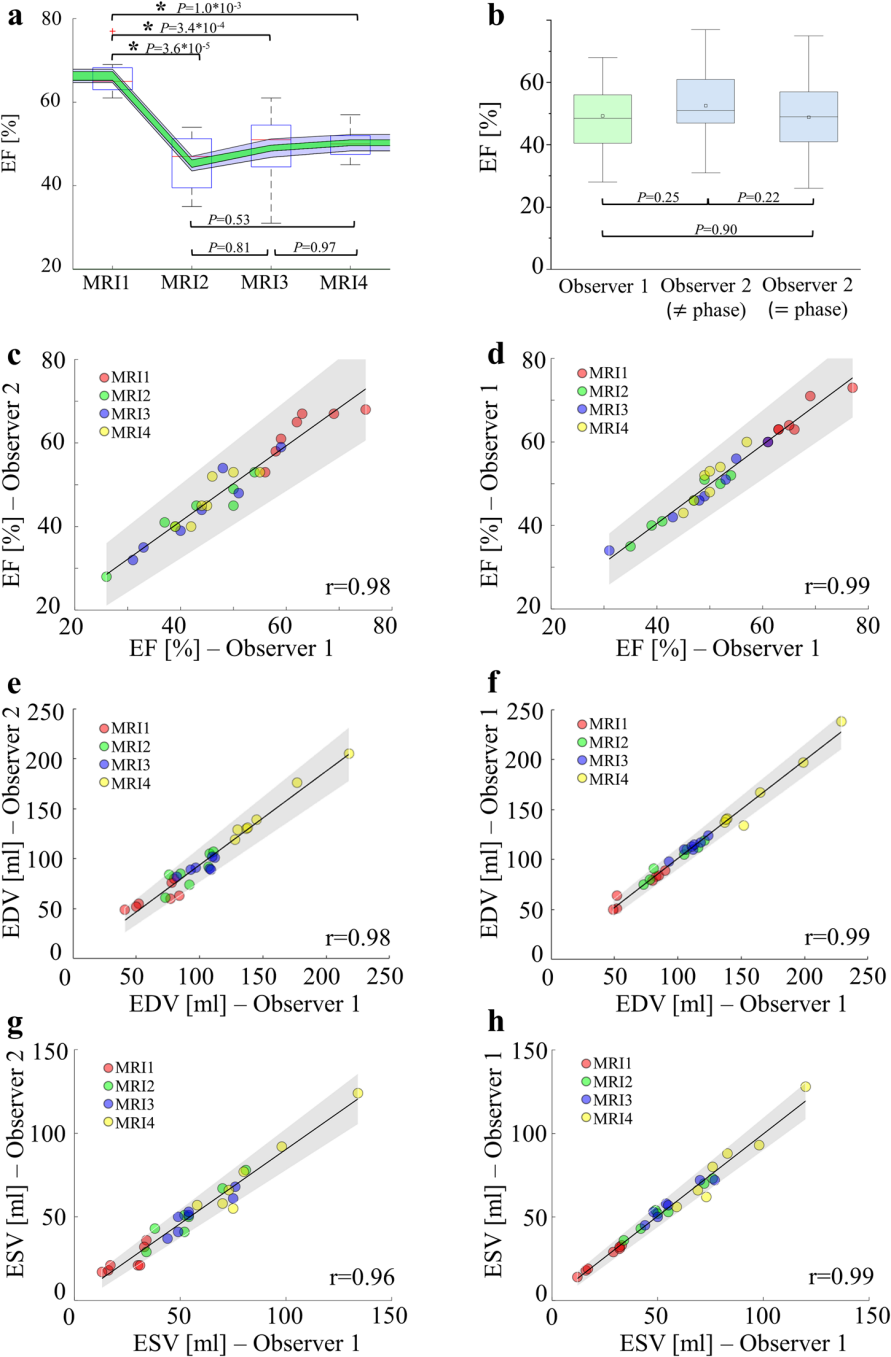
Assessment of cardiac function in a large animal model of acute and chronic infarction. **a** Ejection fraction at baseline (MRI1), acute (MRI2, 4 ± 1 days post MI), sub-acute (MRI3, 12 ± 1 days post MI), and chronic (MRI4, 58 ± 2 days post MI) phases of the study. The asterisk indicates significant differences in a paired t-test (*n* = 7, *p* < 0.05). Green and gray backgrounds illustrate the impact of inter-observer and intra-observer variability, respectively. **b** Influence of the selection of end-diastole and end-systole on derived ejection fraction compared to another observer. Selecting identical phases for end-diastole and end-systole (“harmonization”) shows that manual segmentation between the two observers matches well for all time points. Statistical differences were tested using a paired t-test (*n* = 28, *p* < 0.05). **c**, **e**, **g** Correlation of EF, EDV, and ESV values determined by two different Observers. **d**, **f**, **h** Correlation of EF, EDV, and ESV values determined by the same Observer. Gray shading in (**c**–**h)** indicates 95% confidence intervals of the fit. Color-coding in (**c**–**h**) corresponds to measurement time points prior to and post MI. Boxes in boxplots depict 25th to 75th percentiles, while medians are marked as horizontal line. Whiskers extend to the most extreme data points and outliers are indicated by red pluses. Sample size in boxplots for (**a**) is *n* = 7 each. Sample size in boxplots for (**b**) was *n* = 28 each. EF ejection fraction, EDV end-diastolic volume, ESV end-systolic volume, MI myocardial infarction, MRI magnetic resonance imaging.

Compared to the baseline ejection fraction of (66 ± 5)%, MI decreased ejection fraction in the acute, the sub-acute and the chronic phase to (45 ± 7)%, (49 ± 9)%, and (50 ± 4)%, respectively (Fig. 5). These reductions compared to baseline were found to be statistically significant, whereas differences between acute, sub-acute, and chronic phase were not statistically significant (Fig. 5a). Mean values for metrics derived from the CINE data are listed in Table 2. EF, end-diastolic volume (EDV), and end-systolic volume (ESV) values for individual scans are listed in Supplementary Tables 2–4, and physiological data like blood pressure and heart rate in Supplementary Table 5. ESV showed a negative correlation (*r* = −0.71) with ejection fraction, whereas EDV only slightly (*r* = −0.45) correlated with ejection fraction. These results support the stated wall motion abnormality during contraction in mid-cavity and apical slices of the LV.

**Table 2:** Parameters of left ventricular cardiac function derived from CINE MRI throughout the study

Metric	MRI1, baseline (Prior to MI)	MRI2, acute (4 ± 1 days pMI)	MRI3, sub-acute (12 ± 1 days pMI)	MRI4, chronic (58 ± 2 days pMI)
EF [%]	66 ± 5	45 ± 7	49 ± 9	50 ± 4
SV [ml]	46 ± 9	43 ± 8	54 ± 11	83 ± 15
ESV [ml]	25 ± 8	54 ± 15	57 ± 12	84 ± 23
EDV [ml]	66 ± 16	93 ± 15	102 ± 10	153 ± 30
ED mass [g]	81 ± 8	104 ± 8	113 ± 6	157 ± 18

*MI* myocardial infarction, *pMI*: post myocardial infarction, *EF* ejection fraction, *SV* stroke volume, *ESV* end-systolic volume, *EDV* end-diastolic volume, *ED* end-diastolic.

Mean values ± a single standard deviation.

The high quality of the CINE images enabled the achievement of a high reproducibility in segmentation and resulting ejection fraction (see Fig. 5a). Without “phase harmonization” (matched cardiac phases for end-systole and end-diastole between the two observers) inter-observer variability was systematically increased compared to ejection fraction post “phase harmonization”. The latter procedure resulted in excellent agreement between the results of both observers (Fig. 5b). Overall CoVs for inter-observer variability prior to and post harmonization were 5.8% and 3.8%, with inter-observer pooled SD of 2.8% and 1.9%, respectively. Based on the low inter-observer pooled standard deviation (1.9%) a clinically relevant difference in LV EF (3%, further data in Supplementary Table 6) can already be detected in a sample size of *n* = 9 animals per group (power of 90% at the 5% significance level). CoVs (post harmonization) for MRI1, MRI2, MRI3, and MRI4 were 3.2%, 3.5%, 4.4%, and 3.9%. Pooled SDs for the same measurements were 2.0%, 1.5%, 2.0%, and 1.8%. Figure 5c reveals that ejection fractions determined by our two observers exhibit strong correlation (*r* = 0.97), independent of the measurement time point and therefore, independent of the animal size and health status, and independent of the RF coil used. CoVs for EDV for MRI1, MRI2, MRI3, and MRI4 (post harmonization) were 6.0%, 5.4%, 9.3%, and 5.2%, while CoVs for ESV were 5.5%, 11.0%, 8.9% and 5.8%, respectively. CoV for inter-observer variability in EDV and ESV were 6.5% and 7.8%. Figure 5e, g depict great correlation between EDV (*r* = 0.93) and ESV (*r* = 0.96) values determined by the two observers. Bland-Altman plots for EF, EDV and ESV are illustrated Supplementary Fig. 7 as are the metrics myocardial mass and stroke volume (SV) in Supplementary Fig. 8.

CoV and pooled SD for intra-observer variability were 2.0% and 1.0% and ejection fraction values derived from the two segmentations showed strong correlation (*r* = 0.98). CoVs (intra-observer) for MRI1, MRI2, MRI3, and MRI4 were 2.1%, 1.8%, 1.4%, and 1.4%, respectively. Pooled SDs for the same measurements were 2.0%, 1.5%, 2.0%, and 1.8%. With the lower inter-observer pooled SD (1%) a clinically relevant difference in LV EF (3% further data in Supplementary Table 4) can already be detected in a sample size of *n* = 3 animal per group (power of 90% at the 5% significance level). Like inter-observer variability, Fig. 5d shows that the intra-observer variability was independent of the animal size, health status, and the RF coil used. CoVs for EDV for MRI1, MRI2, MRI3, and MRI4 (post harmonization) were 1.8%, 3.6%, 1.7%, and 4.2%, while CoVs for ESV were 2.6%, 3.0%, 4.0% and 5.0%, respectively. CoV for inter-observer variability in EDV and ESV were 2.8% and 3.6%. Figure 5f, h depict excellent correlation between EDV (*r* = 0.99) and ESV(*r* = 0.99) values determined by the same observer. Bland-Altman plots for EF, EDV, and ESV are illustrated in Supplementary Fig. 7 as are the metrics myocardial mass and SV in Supplementary Fig. 8.

### Scar quantification based on LGE

**Fig. 6:**
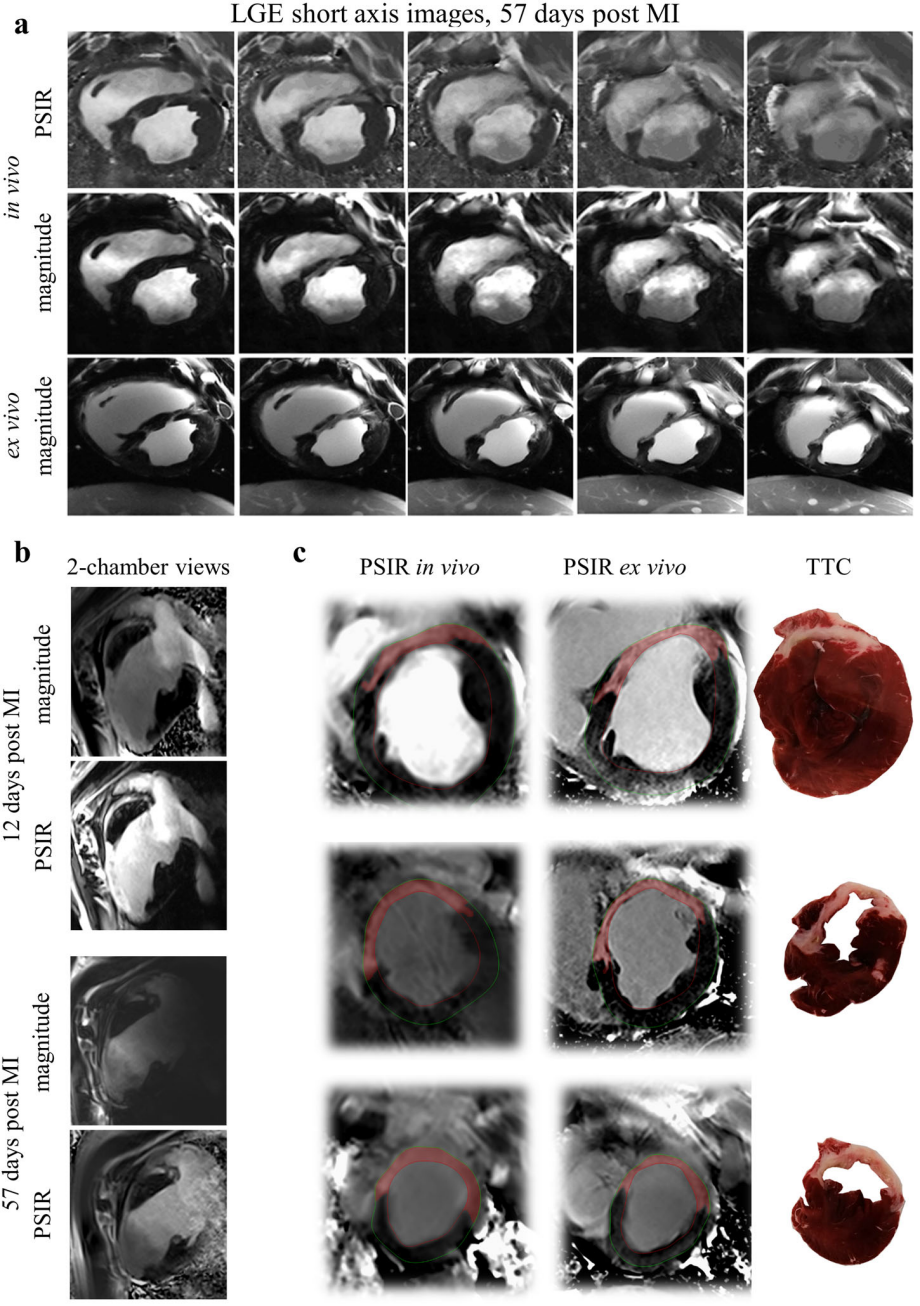
7 T late gadolinium enhancement in large animals (30–82 kg) with acute and chronic infarction. **a** Representative short axis LGE images both in vivo and ex vivo 57 days post MI. Images demonstrate great correlation between in vivo and ex vivo measurements, in particular for PSIR data. Images are windowed for scar visibility. **b** 2-chamber magnitude and PSIR LGE images 12 and 57 days post MI. **c** Comparison of in vivo and ex vivo LGE as well as TTC data for a basal, mid-cavity, and an apical slice. LGE images were selected to visualize individual artifacts such as signal enhancement in the lateral wall due to insufficient inversion or streaking artifacts due to trigger inconsistencies in the mid-cavity slice. Corresponding TTC images, particularly the basal slice, showcase the difficulty for segmentation regarding endo- and epicardial borders for MI quantification. LGE late gadolinium enhancement, MI myocardial infarction, PSIR phase sensitive inversion recovery, TTC triphenyl tetrazolium chloride.

LGE imaging of the short axis enabled a clear depiction of scar tissue at all time points after infarct induction, while baseline scans showed that adequate magnetization inversion and thus nulled signal in the myocardium throughout the LV was achieved. In individual scans at later stages of the study (MRI 4) magnetization inversion was not fully achieved in very basal slices outside the infarcted area (Supplementary video 2). Representative mid-cavity short axis images for in vivo and ex vivo scans are displayed in Fig. 6a. Contrast agent-induced signal enhancement is clearly visible in the septum and the anterior wall post MI. Image quality achieved in in vivo measurements, particularly PSIR data, enabled scar delineation and the depiction of fine details, correlating well with high-resolution LGE images acquired ex vivo (Fig. 6). For the assessment of transmural extension of scar tissue, LGE was also acquired in 2- and 4-chamber views. Representative 2-chamber views are shown in Fig. 6b. In addition to information on the transmural extension of the scar, these images also demonstrate that adequate magnetization inversion was achievable from base to apex. While the scan 12 days post MI exhibits good signal for the atrium as well, this is not the case for the scan 57 days post MI. Validation of infarct sizes measured in vivo was performed via ex vivo LGE measurements as well as TTC staining both post MRI4. Figure 6c shows LGE images of such scans and the matched TTC slices. The large amount of viable tissue (red) in the TTC images complicated delineation of endo- and epicardial borders and thus, affected infarct quantification. Basal LGE images exhibit the above-mentioned signal enhancement in the lateral wall.

**Fig. 7:**
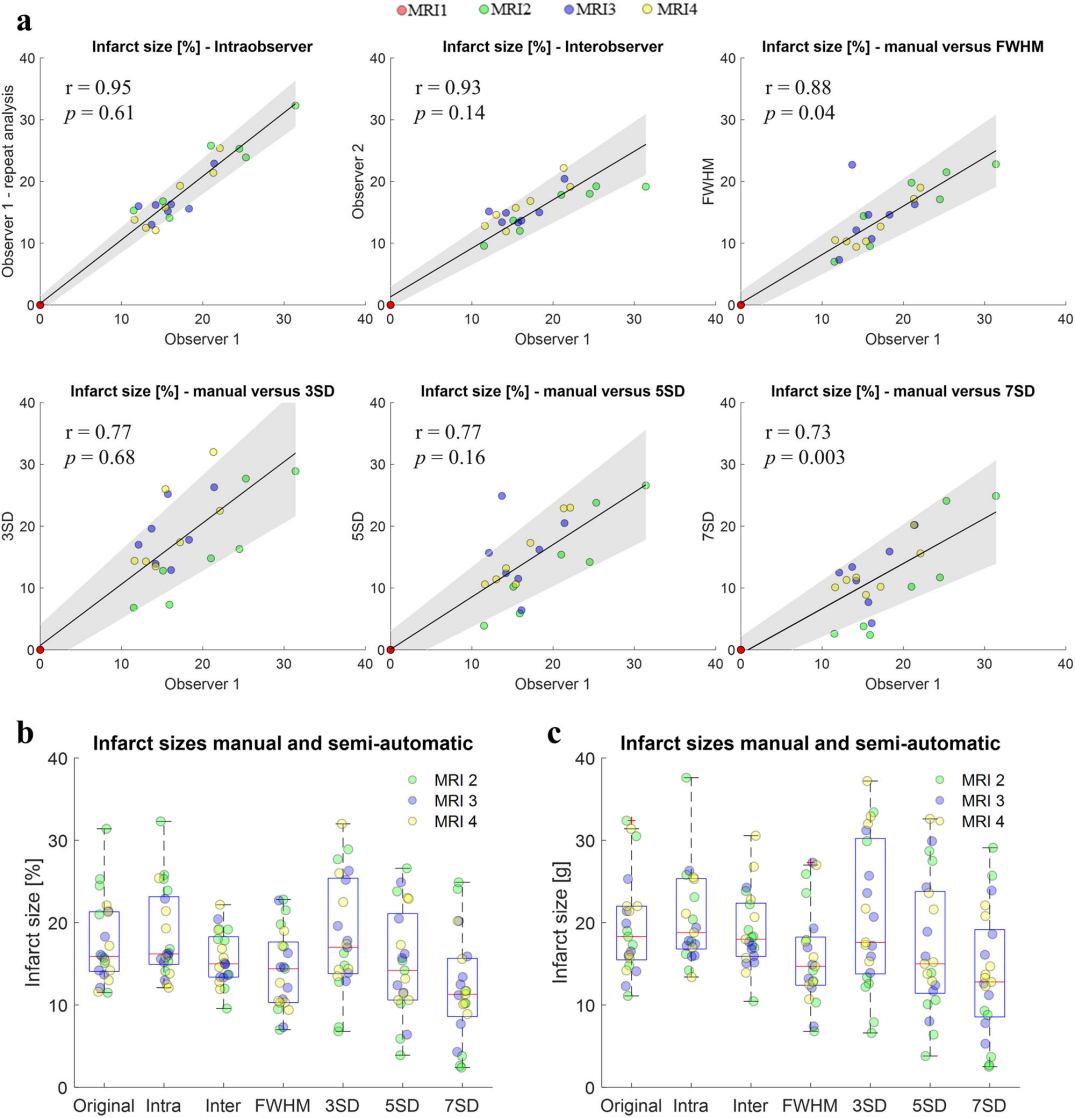
Correlation of manual and semi-automatic infarct quantification based on PSIR images. **a** Correlation plots for intra-observer variability, inter-observer variability, and the semi-automatic methods FWHM, 3SD, 5SD, and 7SD relative to manual segmentation. **b** Boxplot for infarct size [%] distributions for all segmentations. **c** Boxplot for infarct size [g] distributions for all segmentations. Color-coding corresponds to measurement time points prior to and post MI. Significance values are based on paired t-tests (*p* < 0.05) with the original segmentation. Significant differences post Bonferoni correction are marked with an asterisk. MRI indices refer to baseline (MRI1), acute (MRI2, 4 ± 1 days post MI), sub-acute (MRI3, 12 ± 1 days post MI), and chronic (MRI4, 58 ± 2 days post MI) phases of the study. Boxes in boxplots depict 25th to 75th percentiles, while medians are marked as horizontal line. Whiskers extend to the most extreme data points and outliers are indicated by red pluses. Sample size in boxplots was *n* = 21 each. Gray shading in a indicates 95% confidence intervals of the fit. Color-coding in (**a**–**c**) corresponds to measurement time points prior to and post MI. FWHM full width half maximum, MI myocardial infarction, MRI magnetic resonance imaging, PSIR phase sensitive inversion recovery, SD standard deviation.

Overall image quality enabled high reproducibility in segmentation of scar tissue. Infarct sizes determined based on magnitude images were systematically lower than based on PSIR images (*p* < 0.001 for MI in % and g). The following results thus refer to infarct sizes derived from PSIR images unless otherwise stated. Figure 7 shows infarct sizes based on manual as well as semi-automatic segmentation approaches as well as their correlation to manual segmentation. Highest correlations (*r* = 0.95 and *r* = 0.93) were found for manual segmentation (intra- and inter-observer variability). While all semi-automatic approaches achieved high ICCs regarding intra-observer variability (0.92–0.99, see Supplementary Table 7), the FWHM method performed best compared to manual segmentation (*r* = 0.88). Infarct sizes determined based on the standard deviation method showed decreasing correlation (*r* = 0.77, *r* = 0.77, *r* = 0.73) with increasing standard deviation (3SD, 5SD, 7SD) compared to manual segmentation. With median values of 15.0% and 14.4%, Observer 2 and the FWHM method slightly underestimated infarct sizes compared to the original manual segmentation (median: 15.9%) as shown in Fig. 7b. This underestimation was less distinct in inter-observer variability of the infarct size in g tissue mass (18.3 g versus 18.0 g) but persisted for the FWHM method (14.7 g, see Fig. 7c). Infarct sizes derived based on the standard deviation method showed higher variance compared to the other methods as shown in Fig. 7b, c. Values for individual scans are listed in Supplementary Table 8. CoV and ICC for intra-observer variability in infarct sizes [g] were 6.9% and 0.96. CoV and ICC for inter-observer variability were 14.4% and 0.87, while the FWHM method provided the lowest CoV (17.9%, ICC: 0.70) out of the semi-automatic approaches. On average, infarct sizes at time points of MRI2, MRI3, and MRI4 were (23.3 ± 7.6)%, (18.9 ± 4.3)%, and (18.7 ± 3.5)%, which corresponds to (20.4 ± 7.6)g, (17.6 ± 4.6)g, and (20.6 ± 5.8)g, respectively.

### Scar quantification based on TTC

**Fig. 8:**
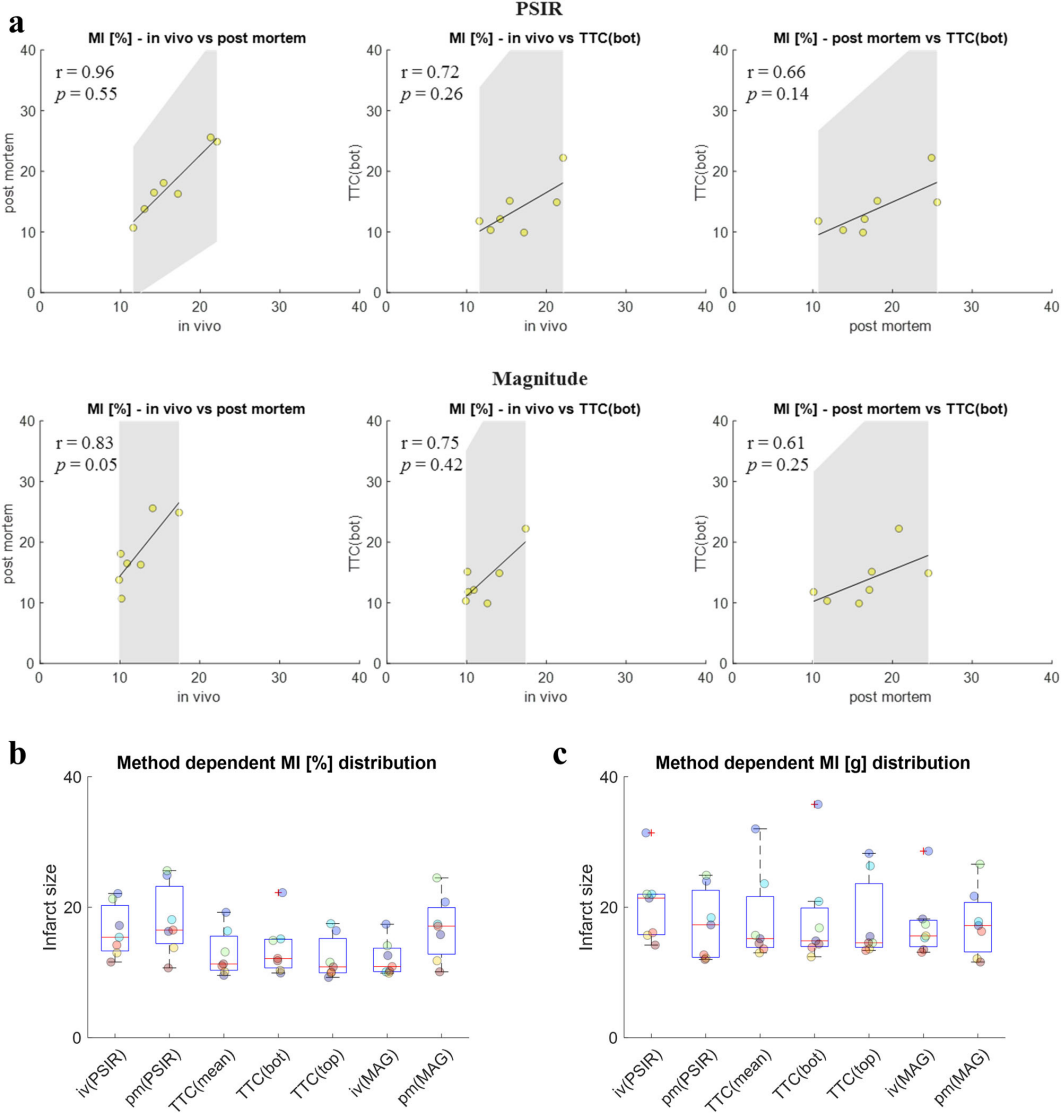
Correlation of infarct sizes derived from in vivo and ex vivo LGE images as well as TTC staining. **a** Correlation plots for infarct sizes in % derived from PSIR and magnitude LGE images. r-values denote Pearson correlation coefficients (*n* = 7) and significance values are based on a Bonferroni corrected paired t-test (*n* = 7). **b** Method dependent infarct sizes [%] over the whole LV volume for all seven animals. **c** Method dependent infarct sizes [g] over the whole LV volume for all seven animals. “iv” denotes in vivo scans, “pm” denotes post mortem scans. TTC(mean), TTC(bot), and TTC(top) refer to the way infarct sizes were determined from TTC images. Values for TTC(bot) were derived from the bottom view of the slice (line of sight from apex to base), values for TTC(top) from the top view (line of sight from base to apex), and values for TTC(mean) as the mean of both views. Color-coding in (**b**, **c**) corresponds to the different animals. Boxes in boxplots depict 25th to 75th percentiles, while medians are marked as horizontal line. Whiskers extend to the most extreme data points and outliers are indicated by red pluses. Sample size in boxplots was *n* = 7 each. Gray shading in (**a**) indicates 95% confidence intervals of the fit. Color-coding in (**a**) corresponds to the measurement time point post MI (MRI4). Color-coding in (**b**–**c**) corresponds to the individual animals. iv in vivo, pm post mortem, LGE late gadolinium enhancement, MAG magnitude, MI myocardial infarction, PSIR phase sensitive inversion recovery, TTC triphenyl tetrazolium chloride.

Infarct sizes derived from high-resolution ex vivo LGE acquired during MRI4 and TTC staining post mortem were (18.0 ± 5.5)% and (18.9 ± 6.8)g and (13.8 ± 4.2)% and (18.4 ± 8.1)g. Figure 8a depicts respective correlation plots for infarct sizes in % derived from both PSIR and magnitude images (Individual data is listed in Supplementary Table 9). Infarct sizes for TTC data in these plots is based on the bottom view of the TTC image, meaning with line of sight from apex to base. Correlation plots for the line of sight from base to apex and also the mean of the two are depicted in Supplementary Fig. 9. Correlations between in vivo and ex vivo LGE data for both PSIR (*r* = 0.96) and magnitude images (*r* = 0.83) were stronger than between in vivo and TTC data (*r* = 0.72, *r* = 0.75). Infarct sizes in % derived from TTC data were found to be systematically lower than in vivo and ex vivo LGE data as shown in Fig. 8b. CoVs for infarct sizes in % based on in vivo and ex vivo LGE data were 6.0% for intra-observer variability and 6.6% for inter-observer variability. Direct comparison of the infarcted tissue in g tissue improved comparability between LGE and TTC data (Fig. 8c). CoVs for infarct sizes in g tissue mass based on in vivo and ex vivo LGE data were 7.3% for intra-observer variability and 5.2% for inter-observer variability.

## Discussion

With cardiac 7 T MRI still being a research rather than a routine imaging modality in large subjects such as large animals and humans, it was paramount to demonstrate that consistent image contrast can be achieved in a wide range of thorax dimensions and animal weights. In this study we used three cine acquisitions with varying flip angles (5° increments) to determine optimal blood tissue contrast in each pig at each examination prior to running the short axis cine stack for cardiac function. We demonstrate that our dedicated RF hardware in combination with such a procedure enables consistent blood tissue contrast in large animals of various sizes and weights (30–82 kg) at 7 T.

The shape of each RF coil was optimized for thorax dimensions in a certain weight range (see Supplementary Fig. 1, coil1: 25–40, coil2: 40–60 kg, coil3: ≥60 kg). The short distances between target tissue and coil elements in combination with dedicated hardware optimizations enabled consistently high SNR, particularly in animals lighter than 60 kg (MRI1, MRI2, and MRI3). The distinctly lower SNR in MRI4 (coil3) is attributed to the bigger distance between the heart and the coil in animals with high body weight. To generate sufficient signal penetration also in the posterior wall of the heart at such a distance, coil elements (coil3) were designed to be 20% bigger than in coil1 and coil2 (see Supplementary Figs. 1, 2), which decreased receive sensitivity as a trade-off. On one hand, a tight allocation of elements leads to more elements receiving signal and therefore, contributing to the combined SNR. This typically makes the RF coil more efficient. On the other hand, closely positioned coil elements result in elevated RF-noise correlation and amplification (higher g-factor)^36^. A detailed assessment of the g-factor and receive characteristics was beyond the scope of this paper, but will be subject to future work. Future developments in RF hardware (32/64 receive channels as well as parallel transmit methodology) will allow us to harness the advantages associated with 7 T even more efficiently, pushing the limits for achievable precision in metrics of cardiac function even further compared to clinical field strengths. This trend will very likely be supported by current and future developments in artificial intelligence^37^, addressing image reconstruction, but more importantly image segmentation^38^ which addresses restrictions regarding operator variability and therefore precision directly.

In CMR, achievable SNR is usually translated into spatial and/or temporal resolution. Other large animal CMR studies performed at 1.5^18–24^ and 3 T^23,25–27^ have acquired cine data with spatial resolutions ranging from 1.3 × 1.3 × 6 mm^3^ to 2.4 × 2.4 × 5 mm^3^ (voxel volumes: 10–29 mm^3^) and 1.2 × 1.2 × 5 mm^3^ to 1.8 × 1.6 × 8 (voxel volumes: 7–23 mm^3^), respectively. Compared to these studies at lower field strength, our CINE data with moderate resolution improved voxel volumes by at least factors of ~4.7 and ~3.1, whereas our high-resolution cine reduced voxel volumes at least by a factor of ~10.6 and 7.1, respectively. Typical spatial resolutions used for LGE imaging at 1.5 T and 3 T range from 0.9 × 1.1 × 5 mm^3^ to 2.5 × 1.8 × 6 mm^3^ (voxel volumes: 4.7–27 mm^3^). It is noteworthy that LGE imaging studies in large animals at 3 T have not leveraged the increased field strengths for higher spatial resolution than in 1.5 T studies. Compared to the mentioned voxel volumes our LGE data improved voxel volumes at least by a factor of ~2.2.

In our study, measurement protocols for both cine and LGE imaging have been set up with two goals in mind; (1) enable consistent image quality, and (2) use image resolutions comparable to or better than clinical imaging in humans. Image resolutions provided in this study should therefore be considered as the lower limits achievable in pigs at 7 T, particularly for smaller animal sizes where dedicated coils enabled very high SNR that could be translated in even higher image resolutions. In humans, particularly in patients, the distance between coil elements and the heart is likely more comparable to larger animals (≥60 kg). In addition, it is unlikely that vendors will develop multiple RF coils dedicated to certain thorax geometries and dimensions, which means that achievable SNR is likely lower compared to pre-clinical studies in younger German landrace pigs or miniature pigs.

Baseline EF ((66 ± 5)%) in our model is comparable to values observed in humans ((67 ± 4.6)%)^39^, whereas values in miniature pig models such as Yucatan ((63 ± 7.5)%)^40^ and Göttingen ((51.6 ± 0.8)%)^41^ miniature pigs appear to be somewhat smaller. Our post-MI EF values are also in good agreement with another longitudinal large animal study (90 min LAD occlusion) in Yucatan miniature pigs where EF values in the acute stage (2 days post MI) were 53 ± 8% and 53 ± 3% in the chronic stage (6 months post MI)^40^. The observed decrease in infarct size from the acute to the chronic stage has been described in other animal models as well^40,42^.

Precision in the assessment of cardiac function is gated by operator variability during acquisition, data quality (resolution, contrast, absence of artifacts), and intra- as well as inter-observer variability in post-processing and data analysis. Many clinical decision-making guidelines rely on the metric of LV EF^43–45^ and for heart failure this metric is critical to both diagnosis and therapy decisions^46,47^. In these instances, higher precision means that smaller changes are more reliably detected with statistical significance, enabling more informed treatment decisions at an earlier stage of treatment response. Additionally, higher precision means that sample sizes required to detect the same changes in metrics of cardiac function can be smaller, supporting the 3R principle for pre-clinical studies and reducing the economic and logistical burden of both pre-clinical and clinical studies, where metrics of cardiac function and also infarct size act as surrogate endpoints.

While there are numerous reproducibility studies for cardiac function in patients^48–53^, such literature reports are scarce for large animal studies. Our data indicates that 7 T cardiac MRI enables high precision measurements in large animals. Coefficients of variation for intra- and inter-observer variability in LV EF and more importantly, pooled SD, were 2.0% and 3.8% as well as 1.0% and 1.9%, respectively. Similar to ref. ^49^. who describes the positive effect of training on inter-observer variability, we see that harmonization of analysis improved inter-observer variability. Selvakumar et al. analyzed reproducibility in EF in an infarction model (90 min LAD occlusion, female Landrace pigs) at 3 T, comparing a free breathing acquisition with a breath hold acquisition^35^. They found that breath holds significantly improved image quality and reproducibility in metrics of cardiac function. Based on these acquisitions they reached CoVs of 2.7% and 4.5% and pooled SDs of 1.5% and 2.6% for intra- and inter-observer variability in LV EF. While our improvements in these metrics appear to be small, they still have significant impact on the sample sizes required to detect a desired change in a metric with high probability, because sample size and pooled SD are related by a square function. A reduction from 1.5% to 1.0% and from 2.6% to 1.9% regarding pooled SD effectively cut sample size requirements in half (assuming 90% power and an α error of 0.05 to detect an absolute change in EF of 5%). Compared to EF, CoVs for EDV and ESV values were higher for both intra- (2.8% and 3.6%) as well as inter-observer (6.5% and 7.8%) variability. While there are no other large animal studies reporting on reproducibility in these metrics, this should be considered when designing large animal studies, particularly with respect to sample size.

Compared to observations in humans (CoV_Intra-observer_: 2.3–5.2%^48,50–52^, CoV_Inter-observer_: 2.9–6.0%^48–53^), we demonstrate high reproducibility in values of EF. Similarly, we show great reproducibility in EDV (CoV_Intra-observer_: 2.6–4.3%, CoV_Inter-observer_: 2.9–5.6%) and ESV (CoV_Intra-observer_: 6.5–10.1%, CoV_Inter-observer_: 6.8–10.5%)^48–53^. It is noteworthy that higher CoVs for ESV compared to EDV are typically found in humans as well. Potentially the biggest influence regarding reproducibility in ESV are papillary muscle and trabeculations, where inclusion or exclusion can lead to differences in ventricular dimensions and function^54^. Our high reproducibility in EF, EDV, and ESV is of particular interest for two reasons (1) animals scanned in the acute and sub-acute phase of MI in this study are still young and heart sizes are considerably smaller compared to human patients. In these cases both imaging and data analysis are considered to be more challenging^55^. (2) images acquired using a steady-state free precession (SSFP) sequence at clinical field strengths have been shown to improve reproducibility in cardiac function compared to data acquired with a spoiled GRE sequence^52,56,57^. While we demonstrate a markedly improved BTC at 7 T (2.6 ± 0.3 at end-diastole and 2.1 ± 0.2 at end-systole) compared to literature reports at 1.5 T (1.5 ± 0.4 at end-diastole and 1.4 ± 0.3 at systole)^57^, further improvements in BTC at 7 T based on a SSFP sequence may be feasible, pushing image quality and reproducibility even more. Ankenbrand et al. have shown that deep learning based approaches for rapid automatic segmentation can be applied to 7 T CINE images^38^. A transfer of the applied methods to preclinical data may enable consistently high reproducibility and rapid data processing in future preclinical 7 T MRI.

With respect to intra-observer variability in infarct sizes [%] derived from in vivo LGE we achieved r_Pearson_: 0.95, CoV: 6.9%, and ICC: 0.96. Respective values for inter-observer variability were r_Pearson_: 0.86, CoV: 14.4%, and ICC: 0.87. Since large animal studies mainly focus on animal model establishment, method development, validation (ex vivo measurements and histology) and the comparison on semi-quantitative approaches for segmentation, rather than reproducibility in vivo, benchmarking our results is difficult. Lenkey et al. ^58^. report ICCs of 0.79–0.86 and 0.82–0.92 for inter and intra-observer variability and ref. ^59^. report ICCs of 0.94–0.97 and 0.94–0.96 for inter and intra-observer variability, respectively. It has to be noted that these reports are based on semi-automatic quantification approaches, while our best results are based on manual infarct segmentation. Compared with the high ICCs reported by Nguyen et al. we achieved comparable ICCs based on FWHM method (0.93–0.96) and standard deviation approaches (0.92–0.99), despite the use of only half the contrast agent dose. In addition, our achieved intra-observer reproducibility based on PSIR and magnitude images (ICC: 0.96–0.99) was also higher than reported in humans (ICC_intraobserver _= 0.95–0.97)^60,61^, despite the above mentioned difference in heart size. Our observation of overestimation of MI sizes using semi-automatic approaches with low standard deviation (<5SD) is in line with other literature reports^58,62^ as is the improved correlation when using the FWHM method^58^.

In this study we complemented in vivo imaging with high-resolution ex vivo LGE imaging in situ where image degradation due to sub-optimal ECG triggering and cardiac motion are eliminated. This allowed us to validate the accuracy and reproducibility of in vivo LGE acquisitions for the chronic stage. Average infarct sizes derived from in vivo and in situ LGE were (16.4 ± 4.0)% and (18.0 ± 5.5)%. Statistical analysis (*r* = 0.96, *p* = 0.55, CoV = 6.6%, Fig. 8a) confirmed excellent correlation between metrics derived from in vivo and in situ measurements by the same observer. In addition, the high reproducibility of in situ LGE volumes measured by intra-observer variability (CoV: 6.0%) as well as inter-observer (CoV: 6.6%) variability emphasizes the good image quality achieved in this validation scan. The remaining differences between LGE derived from in vivo and in situ scans can be attributed to image artifacts in the in vivo scan, partial volume effects, because of the lower image resolution, differences in the contraction state of the heart, as well as slight offsets of the slice position after euthanasia. Comparison to TTC data confirmed good agreement between infarct sizes estimated based on LGE imaging both in vivo and in situ. The fact that infarct sizes in % relative to the total LV myocardium were systematically lower in TTC data than in LGE data can be attributed to the way the tissue was processed post excision in this study. In particular, delineation of healthy myocardium with respect to papillary muscle and partial volume effects at the endo- and epicardial borders was difficult in images of TTC stained tissue (see Fig. 6). This may have led to overestimation of the total LV volume. Distinctly improved correlation (r: 0.92) between infarct sizes determined in vivo and TTC in g tissue mass support this observation.

While we show that dedicated data analysis with respect to the line of sight for TTC data and also the comparison in g tissue mass rather than infarct size fraction relative to the total LV improves comparability between methods, differences do remain. Impacts of slice discontinuity in TTC data and the line of sight are in the range of 1–2% of the total infarct size in each slice (Supplementary Fig. 10). Discontinuity here means the difference in infarct size determined for the bottom and top view of two adjacent slices, which in theory, should be equivalent. Differences may be the result of sawing for tissue processing or just related to segmentation. Line of sight here means the difference in infarct size determined for the bottom and the top view of the same slice. In this study we acquired images of TTC slices from both sides, meaning from the top (line of sight from base to apex) and the bottom (line of sight from apex to base). Infarct areas in these views may be different depending on the 3D distribution of the infarct, which affects infarct sizes derived from MRI (3D method) and TTC data (2D method). Comparisons between LGE and TTC data may thus work better for the comparison of individual matched slices. Such a data analysis may also improve statistical power due to the increased number of data points. With respect to achievable image quality and resolution in ex vivo MRI, we demonstrate that such acquisitions provide suitable data for validation of in vivo results, particularly when applied with dedicated RF hardware at ultrahigh field strengths.

While our achieved image quality in in vivo LGE enabled reproducible results regarding scar size quantification, higher image resolutions are feasible. Reference power and thus magnetization inversion optimization will be required to further improve image quality, independent of animal weight and thorax dimensions. This is particularly true for basal slices in heavier animals where the distance between coil elements and myocardial tissue is largest and difficulties regarding B_1_ efficiency and thus, magnetization inversion at 7 T are highest. In addition, we used a rather low contrast agent dose compared to other large animal studies. A systematic analysis regarding the impact of contrast agent doses may further improve image quality, sensitivity, and precision of LGE imaging at 7 T. Consistent cardiac gating post MI was difficult to achieve in individual scans, despite the use of an acoustic triggering system. With respect to ventilator induced breath holds we found that a waiting period of 5–10 s post ventilation stop was required to prevent residual thorax motion from impacting image quality.

With *n* = 7 the total number of animals studied was small. While our study setup accommodated the increase in animal size very well, and all animals including the RF coils did fit into the 60 cm scanner bore, this may not be the case for animal weights >100 kg. In addition, the achievable SNR in large animals may be limited by increased distance between the RF coil and the target tissue.

Cardiac MRI is highly dependent on the quality of the trigger signal used for cardiac gating. While we found that the vendor ECG which employs a learning-based correction algorithm works equally well as the acoustic triggering system in humans^5^, this was not the case in our large animal scans. A potential reason for this may be the difference in heart and large vessel orientation compared to human anatomy and its influence on the ECG signal via magnetohydrodynamic effect.

While large animal reperfusion injury models have been described for a variety of porcine breeds and MI induction as well as measurement protocols, they were all performed at clinical field strengths^23,40,63,64^. In addition, the lack of data regarding image quality and reproducibility limits comparability between studies performed using the same field strengths. We demonstrate that 7 T cardiac MRI in a large animal model of acute and chronic MI provides high-precision data for translational studies. More importantly, we demonstrate that dedicated RF hardware and measurement protocols enable consistent blood tissue contrast and image quality in longitudinal studies in pigs, despite drastic changes in thorax dimensions and shape. Such data in combination with our results on reproducibility enables informed decisions regarding future large animal studies at 7T. We thus define state-of-the-art procedures and RF hardware for such studies at 7 T.

Preliminary results of a study assessing scan-rescan reproducibility of cardiac function at 7 T indicate that the high blood tissue contrast for the spoiled GRE sequence can be achieved in humans as well, enabling high reproducibility in metrics of cardiac function such as ejection fraction (CoV: 2.8%, ICC: 0.92) and end-diastolic myocardial mass (CoV: 3.7%, ICC: 0.99)^65^. Direct application of our LGE imaging protocol in humans will be limited by the specific absorption rate. However, developments regarding parallel transmit applications and AI methods may help addressing this limitation in order to enable 7 T LGE imaging in a clinical setting. Dedicated infrastructure in combination with a whole-body 7T system will likely enable pre-clinical precision measurements in CMR on a routine basis. Related improvements in the diagnostic assessment and the monitoring of the progression of cardiac disease will then become an indispensable tool for cardiovascular research in animal models, novel diagnostic methods^66^, interventions^67^, drug trials, and theranostics^68^. With respect to the latter two, the potential of increased SNR and spatial resolution in both ^1^H and especially X-nuclei imaging^9,69–71^ may facilitate application of these highly sensitive 7 T MRI techniques on a routine basis. Progress in the generation of targeted and functionalized nanoparticles and activatable MRI bio-sensors^72^ may further support this development. Pre-clinical cardiac ultrahigh field MRI may enable new insights into cardiac metabolism^70^, ion homeostasis^69^, inflammation^71^, tissue function as well as pharmacokinetics of individual drugs. Finally, such studies will provide grounds for methodology advancements towards human 7 T cardiac MRI.

### Supplementary information

Supplementary Information

Description of Additional Supplementary Files

Supplementary Video 1

Supplementary Video 2

Reporting Summary

## Data Availability

All data used is provided within the contents of this manuscript or the supplementary material pdf. Source data for the figures and Tables can be accessed in text format in the supplementary material. Additional data may be provided upon reasonable request to the study’s PI L.M.S. (Schreiber_L@ukw.de), Head of the Department for Cardiovascular Imaging at the Comprehensive Heart Failure Center in Wuerzburg.
