# Supplementary material for: Precision imaging of cardiac function and scar size in acute and chronic porcine myocardial infarction using ultrahigh-field MRI
Source: Commun Med (Lond). 2024 Jul 18;4:146. doi: 10.1038/s43856-024-00559-y (PMC11258271; doi:10.1038/s43856-024-00559-y)
Supplement: Supplementary file 1 — Supplementary Information [file 43856_2024_559_MOESM1_ESM.pdf]

# – SUPPLEMENTARY INFORMATION –

Precision Imaging of Cardiac Function and Scar Size in Acute and Chronic Porcine Myocardial Infarction Using Ultrahigh-Field MRI

[1]David Lohr*, [1]Alena Kollmann, [1]Maya Bille, [1]Maxim Terekhov, [1]Ibrahim Elabyad, [1]Michael Hock, [1]Steffen Baltes, [1,2]Theresa Reiter, [2]Florian Schnitter, [2]Wolfgang Rudolf Bauer, [2]Ulrich Hofmann, [1]Laura Maria Schreiber

[1]Comprehensive Heart Failure Center (CHFC), Chair of Cellular and Molecular Imaging, University Hospital Wuerzburg, Wuerzburg, Germany

[2]Department of Internal Medicine I, University Hospital Wuerzburg, Wuerzburg, Germany

* Corresponding author:
Dr. rer. nat. David Lohr
Chair of Cellular and Molecular Imaging
Comprehensive Heart Failure Center (CHFC)
University Hospital Wuerzburg
Am Schwarzenberg 15
97078 Wuerzburg
Germany
E-Mail: Schreiber_L@ukw.de

Parts of this paper have been presented at the 31st annual meeting of the International Society of Magnetic Resonance in Medicine - 2022

Parts of this paper have been presented at the 32nd annual meeting of the International Society of Magnetic Resonance in Medicine – 2023

Supplemental Methods

Study protocol

Due to variations in animal weight and thorax dimensions, we performed 7 scans with coil 1, 14 scans with coil 2 and 7 scans with coil 3. We found that increasing experience throughout the study severely improved the quality of the initial placement of the RF coil. Within the first 1-2 scans in a certain weight range the coil had to be repositioned more frequently. Afterwards, the RF coil was placed correctly in the first try in >90% of all cases.

As indicated above we analyzed hearts of increasing size over time. The average slice numbers for short axis CINE stacks for MRI 1, MRI 2, MRI 3, and MRI 4 were 11±1, 11±1, 12±1, 13±1, respectively.

Inversion times of 450 ms and 400 ms were used in 45% and 34% of all LGE acquisitions.

Cardiac gating

All sequences were using cardiac gating. Clinically, cardiac gating is achieved using the vendor integrated ECG, but for most animals, the ECG inside the bore could not be used. Possible explanations for this could be related to the difference in skin composition and electrical properties, electrode placement, and differences in the magneto-hydrodynamic effect (major blood vessel positions/orientations). All cardiac gating was therefore performed using an acoustic triggering system. A prior assessment using a stethoscope significantly improved the quality of the initial placement of the acoustic trigger.

MRI protocol *in vivo* - sequence parameters

All CINE acquisitions were based on the vendor sequence "BEAT" using a spoiled gradient echo pulse sequence. Retrospective gating was applied with 8 segments to reconstruct 30 cardiac phases. Arrhythmia detection was set to 300ms and the scan accelerated using R=3 (GRAPPA) and a weak asymmetric echo. Further parameters were TE/TR: 2.92/45.12 ms, echo spacing: 5.6 ms (corresponds to "true TR", the time between successive RF-pulses), FA: optimal, slice thickness: 6 mm, and bandwidth: 914 Hz/Px. The FOV was 340x318 mm with a base resolution of 288, leading to an in-plane resolution of 0.6x0.6 mm^2 after zero-filling interpolation. On average the scan time was 13.3±2.7 ms per slice.The high resolution CINE measurements maintained these parameters where possible. Changes were TE/TR: 3.18/49.52 ms, echo spacing: 6.2 ms, and bandwidth: 893 Hz/Px. The base resolution was set to 400, leading to an in-plane resolution of 0.4x0.4 mm^2 after zero-filling interpolation. On average the scan time was 16.5±3.2 ms per slice.

LGE images were acquired using a phase-sensitive inversion recovery (PSIR) sequence with a non-selective inversion preparation. Images were acquired in diastole. Scans were accelerated using R=3 (GRAPPA) and a weak asymmetric echo. Further parameters were TE/TR: 1.96/560 ms, FA: optimal, slice thickness 6 mm, and bandwidth: 300 Hz/Px. The FOV was 350x306 mm^2 with a base resolution of 256, leading to an in-plane resolution of 0.68x0.68 mm^2 after zero-filling interpolation. On average the scan time was 6.3±2.8 ms per slice.

B_1^+ maps were acquired using a vendor turbo-FLASH pulse sequence with magnetization preparation. Sequence parameters were TE/TR: 1.5/14190 ms, flip angle: 7°, slice thickness 6 mm, and bandwidth: 490 Hz/Px. The FOV was 360x360 mm^2 with a base resolution of 256x256, leading to an in-plane resolution of 0.70x0.70 mm^2 after zero-filling interpolation.

Data analysis

Cardiac function (EF, ESV, EDV, and myocardial mass) were evaluated using Medis MR Suite. All data was manually segmented by two observers for inter-observer variability. All data was also segmented twice by the same observer for intra-observer variability.

LGE was evaluated using Medis MR Suite. All data was manually segmented by two observers for inter-observer variability. All data was also segmented twice by the same observer for intra-observer variability. In addition data was segmented using semi-automatic approaches, which are frequently applied in clinical practice, namely the 3,5, and 7 standard deviations (SD) approach and the full width at half maximum approach. Descriptions of both are detailed in the method section of the manuscript.

Post mortem LGE and TTC

Post mortem LGE was evaluated manually the same way as *in vivo*. For infarct size quantification based on the images of TTC stained myocardial slices, we applied the open source software tool ImageJ (version 1.53k). Analogous to segmentation in Medis MR Suite, endocardial end epicardial borders of the LV were delineated as well as the infarct area. TTC slices were photographed from the top as well as the bottom. Segmentation and infarct quantification was done for both views, subsequently referred to as "top" and "bot".

In order to assess whether discontinuities between slices due to sawing occurred, we subtracted the infarct size from every top view from the bottom view of the slice. Respective difference values were then normalized to the total infarct size to determine the impact of discontinuity on a slice by slice basis (Fig. S4).

The impact of the line of sight (top versus bottom view) on infarct size was assessed in a similar way. We first calculated respective differences in each slice (top view minus bottom view of the same slice) and then normalized the values to the total infarct size again (Fig. S4).

While Medis also provides the infarct size in g directly, this metric had to be calculated for TTC data. Each TTC image was acquire with a ruler aligned next to the tissue. This information was used to estimate the pixel dimension and subsequently the infarct size in g, assuming a tissue density of 1.05 g/cm^3.

Sample size calculations

Sample sizes (n) required to show a clinically meaningful change in EF with a power of 90% and an α error of 0.05 were calculated as:

$$n = f(\alpha, P) * \sigma^2 * \frac{2}{\delta^2} \quad 1$$

where f denotes a factor that depends on the significance level α and the study power P (for α=0.05 and P=0.90, f=10.5), σ is the inter-observer pooled standard deviation and δ is the desired difference to be detected.[1]

Boxplots

Boxes in boxplots depict 25th to 75th percentiles, while medians are marked as horizontal line. Whiskers extend to the most extreme data points and outliers are indicated by red pluses.

Supplemental Tables

Supplemental Table 1: Image quality metrics derived from CINE MRI throughout the study for individual scans. All metrics are shown for the four MRI time points of all n=7 animals. Animal D died during infarct induction and data therefore omitted from data analysis.

animal #	MRI #	dBTC	sBTC	dSNR	sSNR	dSNR_$B_{1,norm}$	sSNR_$B_{1,norm}$
A		2.25	1.80	155	212	222	328
B		2.88	1.66	122	204	134	258
C		2.60	1.63	132	175	194	278
E	1	2.97	1.98	58	77	75	109
F		3.07	2.09	112	117	142	148
G		2.44	1.88	127	138	148	208
H		2.93	2.09	85	105	175	195
A		2.08	1.98	139	139	174	195
B		2.57	2.11	133	154	153	202
C		2.02	2.11	86	96	89	117
E	2	2.49	2.05	98	93	116	124
F		2.46	2.19	37	39	55	62
G		2.49	2.31	109	106	144	154
H		2.26	2.23	85	91	151	170
A		2.23	1.78	113	124	141	173
B		2.42	2.11	89	122	96	153
C		2.76	2.30	69	66	69	79
E	3	2.32	2.03	96	113	131	17
F		2.53	2.26	102	120	153	194
G		2.75	2.10	71	81	132	167
H		2.69	2.25	87	12	159	188
A		2.98	2.17	53	66	97	128
B		2.64	2.11	49	62	57	84
C		2.52	2.14	54	63	74	95
E	4	2.52	2.01	45	57	50	73
F		2.33	2.23	44	45	60	66
G		3.03	2.21	76	95	107	135
H		2.94	2.36	69	75	105	114

Legend: dBTC: diastolic blood tissue contrast, sBTC: systolic blood tissue contrast, dSNR: diastolic SNR, sSNR: systolic SNR, dSNR_$B_{1,norm}$: diastolic SNR normalized to flip angle, sSNR_$B_{1,norm}$: systolic SNR normalized to flip angle, FA: average flip angle in the anterior wall derived from transversal B_1 maps, $D_{C\text{-}LV}$: distance from coil to LV center

Supplemental Table 2: Ejection fraction derived for individual scans in order to assess intra- and inter-observer variability. All metrics are shown for the four MRI time points of all n=7 animals. Animal D died during infarct induction and data therefore omitted from data analysis.

		ejection fraction - EF [%]			
**animal #**	**MRI #**	**observer 1**	**observer 2 (1st segmentation)**	**observer 2 (2nd segmentation)**	**observer 2 (after harmonization)**
A		53	61	60	56
B		65	63	63	62
C		61	63	63	59
E	1	67	69	71	69
F		68	77	73	75
G		44	49	47	44
H		58	66	63	58
A		28	35	35	26
B		41	41	41	37
C		40	39	40	39
E	2	45	52	50	43
F		53	54	52	54
G		49	47	46	50
H		45	49	51	50
A		44	49	47	44
B		35	43	42	33
C		32	31	34	31
E	3	39	48	46	40
F		54	55	56	48
G		59	61	60	59
H		48	53	51	51
A		45	50	48	45
B		40	47	46	39
C		40	45	43	42
E	4	52	49	52	46
F		53	57	60	55
G		45	50	53	44
H		53	52	54	50

Supplemental Table 3: End-diastolic volume derived for individual scans in order to assess intra- and inter-observer variability. All metrics are shown for the four MRI time points of all n=7 animals. Animal D died during infarct induction and data therefore omitted from data analysis.

		end-diastolic volume - EDV [ml]			
**animal #**	**MRI #**	**observer 1**	**observer 2 (1st segmentation)**	**observer 2 (2nd segmentation)**	**observer 2 (after harmonization)**
A	1	76	83	83	78
B		60	80	79	77
C		80	85	84	80
E		55	52	64	52
F		52	52	51	50
G		49	49	50	41
H		63	90	89	84
A	2	105	116	112	108
B		107	121	119	111
C		85	81	91	85
E		74	105	105	92
F		61	73	75	73
G		84	78	80	76
H		92	107	110	107
A	3	91	105	110	97
B		101	124	124	112
C		90	112	110	108
E		82	93	98	82
F		89	111	113	93
G		92	113	115	109
H		89	118	117	110
A	4	130	165	167	137
B		205	229	238	218
C		131	139	141	138
E		119	137	137	128
F		129	138	140	130
G		139	152	134	145
H		176	199	197	177

Supplemental Table 4: End-diastolic volume derived for individual scans in order to assess intra- and inter-observer variability. All metrics are shown for the four MRI time points of all n=7 animals. Animal D died during infarct induction and data therefore omitted from data analysis.

		end-systolic volume - ESV [ml]			
**animal #**	**MRI #**	**observer 1**	**observer 2 (1st segmentation)**	**observer 2 (2nd segmentation)**	**observer 2 (after harmonization)**
A	1	39	33	33	34
B		21	29	29	30
C		32	32	31	33
E		18	16	18	16
F		17	12	14	13
G		21	17	19	17
H		21	32	32	31
A	2	78	76	73	81
B		67	72	70	70
C		51	49	54	52
E		49	50	52	52
F		21	34	36	34
G		43	42	43	38
H		50	55	53	54
A	3	51	54	58	54
B		68	70	72	76
C		61	77	72	75
E		50	43	46	49
F		41	50	56	49
G		37	44	45	44
H		53	55	57	54
A	4	55	83	88	75
B		124	120	128	134
C		77	76	80	80
E		58	69	66	70
F		57	59	56	58
G		66	73	62	73
H		92	98	93	98

Supplemental Table 5: Physiological parameters during MRI examinations for individual scans. Blood pressure was measured prior to MR scans in the preparation room adjacent to the MR system, while the animal was in supine position. Heart rate data was extracted from DICOM header information of our CINE scans. The heart rate was calculated using the RR-interval. Animal weight was measured early in the morning on measurement days prior to animal preparation. All metrics are shown for the four MRI time points of all n=7 animals. Animal D died during infarct induction and data therefore omitted from data analysis.

animal #	MRI #	weight [kg]	blood pressure [mmHG]	RR-interval [ms]	heart rate [bpm]
A		35	107/48	855	70
B		33	80/56	944	64
C		43	86/18	977	61
E	1	30	92/27	826	73
F		38	90/18	769	78
G		42	65/16	885	68
H		38	73/23	920	65
A		41	84/27	1080	56
B		36	87/22	1184	51
C		46	110/36	988	61
E	2	38	88/22	706	75
F		43	86/70	658	91
G		45	68/22	843	71
H		51	64/12	726	83
A		42	105/26	1142	53
B		41	80/24	1277	47
C		49	82/20	689	87
E	3	40	89/21	1011	59
F		50	89/22	903	66
G		48	100/79	931	64
H		53	109/33	1102	54
A		71	98/29	1188	51
B		70	88/43	1402	43
C		81	101/23	1102	54
E	4	71	71/24	1355	44
F		82	89/35	917	65
G		81	92/29	1354	44
H		74	75/31	921	65

Supplemental Table 6: Impact of reproducibility on sample size requirements for the detection of changes in ejection fraction prior to and post myocardial infarction. Sample sizes are calculated for a 90% power and an α error of 0.05. Samples sizes are always rounded up.

	inter-observer (post harmonization)		intra-observer	
	SD [%]	**sample size [n]**	SD [%]	**sample size [n]**
5% absolute change in LV EF				
MRI1 (baseline)	2.00	**4**	1.40	**2**
MRI2 (acute)	1.48	**2**	0.83	**1**
MRI3 (sub-acute)	1.96	**4**	0.70	**1**
MRI4 (chronic)	1.81	**3**	0.70	**1**
3% absolute change in LV EF				
MRI1 (baseline)	2.00	**10**	1.40	**5**
MRI2 (acute)	1.48	**6**	0.83	**2**
MRI3 (sub-acute)	1.96	**9**	0.70	**2**
MRI4 (chronic)	1.81	**8**	0.70	**2**
1% absolute change in LV EF				
MRI1 (baseline)	2.00	**84**	1.40	**42**
MRI2 (acute)	1.48	**46**	0.83	**15**
MRI3 (sub-acute)	1.96	**81**	0.70	**11**
MRI4 (chronic)	1.81	**69**	0.70	**11**

Legend: LV EF: left ventricular ejection fraction, SD: pooled standard deviation. Bold formatting to emphasize key information.

Supplemental Table 7: Intra-observer variability for manual and semi-automatic infarct quantification approaches. Values in % denote infarct size relative to total LV mass and g denotes infarct size in g tissue mass.

**Intra-observer**	**CoV**	**ICC**
MAG manual %	3.9%	0.987
MAG manual g	6.2%	0.975
PSIR manual %	6.9%	0.959
PSIR manual g	10.1%	0.931
MAG FWHM %	9.5%	0.959
MAG FWHM g	10.4%	0.939
PSIR FWHM %	12.7%	0.946
PSIR FWHM g	15.2%	0.925
MAG 3 SD %	5.5%	0.989
MAG 3 SD g	4.0%	0.991
PSIR 3 SD %	16.6%	0.926
PSIR 3 SD g	22.3%	0.917
MAG 5 SD %	10.1%	0.966
MAG 5 SD g	11.1%	0.917
PSIR 5 SD %	8.5%	0.976
PSIR 5 SD g	9.0%	0.969
MAG 7 SD %	7.2%	0.982
MAG 7 SD g	6.1%	0.961
PSIR 7 SD %	12.2%	0.972
PSIR 7 SD g	13.6%	0.957

Legend: MAG: data based on magnitude images, PSIR: values based on phase sensitivity recovery images, CoV: coefficient of variation, ICC: intra class correlation coefficient.

Supplemental Table 8: Comparison of manual and semi-automatic infarct quantification. "Original" data denotes scar sizes based on manual segmentation of observer AK. "Intra" denotes values based on the manual repeat segmentation of observer AK. "Inter" denotes values based on the manual segmentation of DL. The remaining columns denote values derived using semi-automatic approaches FWHM, 3SD, 5SD, and 7SD.

Scan / animal	Original	Intra	Inter	FWHM	3SD	5SD	7SD
				**Scar size in [%]**			
**2 / A**	11,5	15,3	9,6	7,0	6,8	3,9	2,6
**2 / B**	15,9	14,1	12,0	9,5	7,3	5,9	2,4
**2 / C**	15,1	16,8	13,7	14,4	12,8	10,2	3,8
**2 / E**	21,0	25,8	17,8	19,8	14,8	15,4	10,2
**2 / F**	24,5	25,3	18,0	17,1	16,3	14,2	11,7
**2 / G**	25,3	23,9	19,2	21,5	27,7	23,8	24,1
**2 / H**	31,4	32,3	19,2	22,8	28,9	26,6	24,9
**3 / A**	15,7	15,2	13,3	14,6	25,2	11,5	7,7
**3 / B**	16,1	16,3	13,7	10,7	12,9	6,4	4,3
**3 / C**	13,7	13,0	13,4	22,7	19,6	24,9	13,4
**3 / E**	12,1	16,0	15,1	7,3	17,0	15,7	12,5
**3 / F**	14,2	16,2	14,9	12,1	13,9	12,4	11,2
**3 / G**	21,4	22,9	20,4	16,3	26,3	20,5	20,2
**3 / H**	18,3	15,6	15,0	14,6	17,8	16,2	15,9
**4 / A**	17,2	19,3	16,8	12,7	17,4	17,3	10,2
**4 / B**	22,1	25,4	19,1	19,0	22,5	23,0	15,6
**4 / C**	15,4	15,8	15,7	10,3	26,0	10,6	8,9
**4 / E**	21,3	21,4	22,2	17,2	32,0	22,9	20,2
**4 / F**	13,0	12,5	14,6	10,3	14,3	11,4	11,3
**4 / G**	14,2	12,1	11,9	9,4	13,5	13,2	11,7
**4 / H**	11,6	13,8	12,8	10,5	14,4	10,6	10,1
				**Scar size in g**			
**2 / A**	11,1	14,2	10,5	6,8	6,6	3,8	2,5
**2 / B**	17,3	16,2	17,0	10,3	7,9	6,4	2,7
**2 / C**	14,8	17,7	19,1	14,2	12,6	10,1	3,7
**2 / E**	19,0	23,1	18,3	17,9	13,4	13,9	9,3
**2 / F**	18,3	20,4	18,2	12,9	12,2	10,6	8,8
**2 / G**	30,5	25,8	22,2	25,9	33,4	28,7	29,1
**2 / H**	32,4	37,6	23,9	23,6	29,9	27,5	25,7
**3 / A**	16,0	16,0	15,2	14,9	25,7	11,7	7,8
**3 / B**	19,9	19,4	17,7	13,2	15,9	8,0	5,3
**3 / C**	16,5	15,9	17,2	27,3	23,6	29,9	16,1
**3 / E**	12,3	17,2	16,0	7,4	17,2	15,9	12,6
**3 / F**	14,1	17,4	15,1	12,1	13,9	12,4	11,2
**3 / G**	25,3	26,3	24,3	19,3	31,2	24,3	23,9
**3 / H**	21,4	17,8	16,8	17,0	20,7	18,9	18,6
**4 / A**	21,4	25,3	22,8	15,8	21,7	21,6	14,7
**4 / B**	31,4	40,6	30,6	27,0	32,0	32,6	22,1
**4 / C**	22,0	21,1	20,7	14,7	37,2	15,2	12,8
**4 / E**	22,0	25,5	26,8	17,7	32,9	23,6	20,8
**4 / F**	15,7	17,0	18,0	12,5	17,3	13,8	13,7
**4 / G**	16,1	13,4	13,9	10,7	15,3	15,0	13,3
**4 / H**	14,2	18,8	15,7	12,8	17,6	12,9	12,3

Legend: FWHM: full width half maximum, SD: standard deviation

Supplemental Table 9: Method dependent infarct sizes over the whole LV volume for all seven animals. "iv" denotes *in vivo* scans, "pm" denotes *post mortem* scans. TTC(mean), TTC(bot), and TTC(top) refer to the way infarct sizes were determined from TTC images. Values for TTC(bot) were derived from the bottom view of the slice (line of sight from apex to base), values for TTC(top) from the top view (line of sight from base to apex), and values for TTC(mean) as the mean of both views.

**Animal**	**iv (PSIR)**	**pm (PSIR)**	**TTC (Mean)**	**TTC (Top)**	**TTC (Bot)**	**iv (MAG)**	**pm (MAG)**
**Infarct size in [%]**							
A	17,2	16,3	9,6	9,9	9,2	12,6	15,8
B	22,1	24,9	19,2	22,2	16,4	17,4	20,8
C	15,4	18,1	16,4	15,1	17,5	10,1	17,4
E	21,3	25,6	13,1	14,9	11,6	14,1	24,5
F	13,0	13,8	10,1	10,3	9,9	9,9	11,8
G	14,2	16,5	11,0	12,1	10,0	10,9	17,1
H	11,6	10,7	11,3	11,8	10,8	10,2	10,1
**Infarct size in [g]**							
A	21,4	17,3	15,2	14,8	15,5	18,2	17,2
B	31,4	24,0	32,0	35,8	28,3	28,6	21,7
C	22,0	18,4	23,6	20,9	26,3	15,3	17,8
E	22,0	24,9	15,7	16,8	14,5	17,4	26,6
F	15,7	12,2	13,0	12,4	13,6	15,6	12,1
G	16,1	12,7	13,6	13,8	13,4	13,1	16,3
H	14,2	12,0	14,5	14,4	14,5	13,5	11,6

Legend: iv: *in vivo*, MAG: magnitude, pm: *post mortem*, PSIR: phase sensitivity recovery images

Supplemental Figures

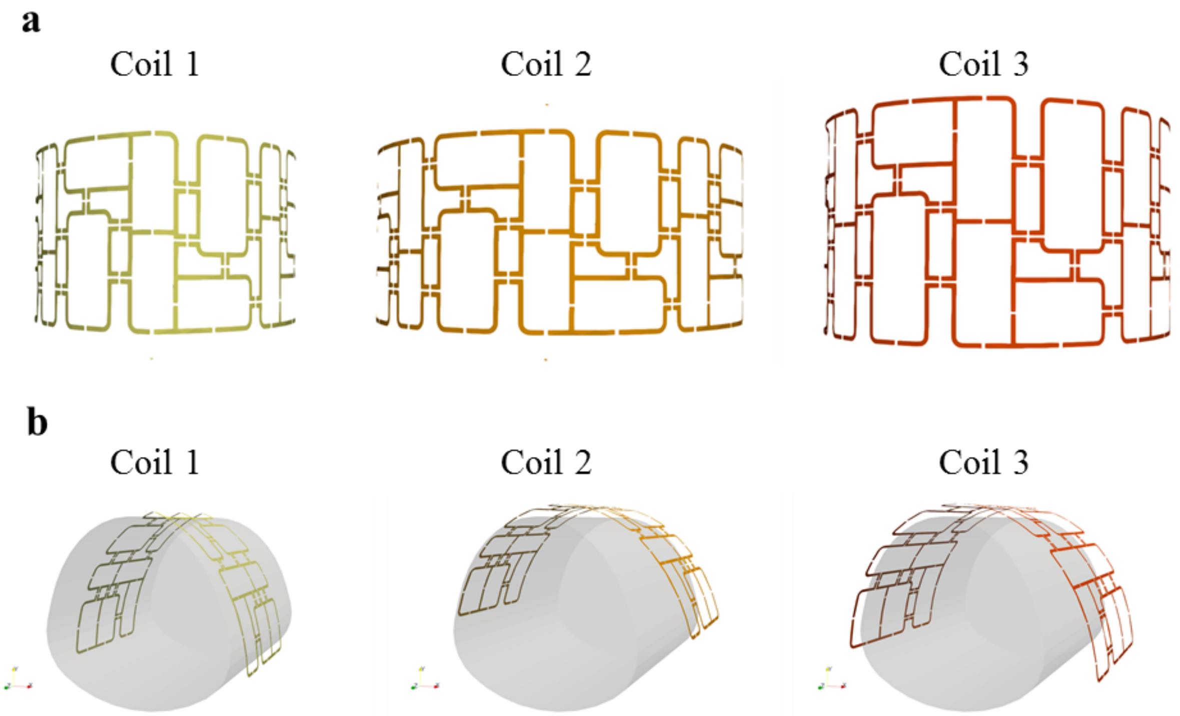

Supplemental Figure 1: Similarities and differences between RF coils 1-3. Layouts for RF-coils and respective conductors as well as a pig thorax phantom are demonstrated with consistent scale. **a:** Top view of the elements in coils 1-3. Coil 1 and coil 2 have the same size for all elements, while elements in coil 3 are 20% bigger to improve penetration. **b:** Angular view including the volume of a customized phantom to illustrate the curvature. The curvature was selected based on the thorax shape in animals of the respective target weight, aiming to minimize distances between the thorax and the coil at all points. Differences in the curvature of coils 1-3 demonstrate how a 60-day growth period of German Landrace pigs effects thorax shape and size.

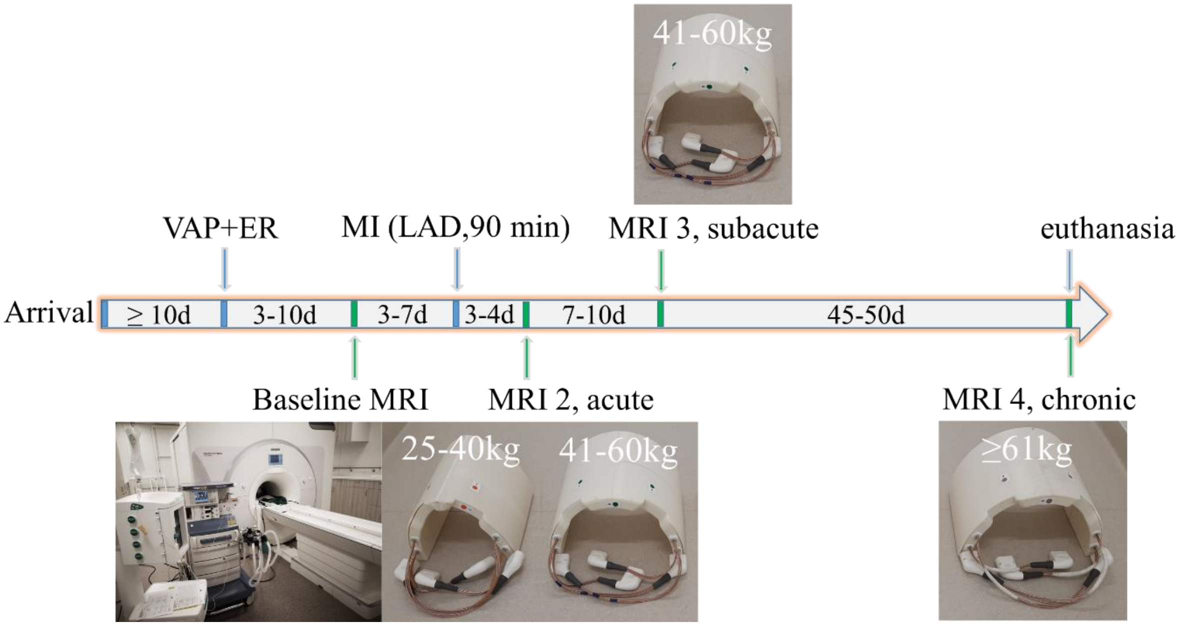

Supplemental Figure 2: Study protocol for ultrahigh field cardiac MRI in a large animal model of acute and chronic infarction. There are 4 MRI scans for each animal included in the study. One baseline MRI prior to induction of MI (proximal LAD occlusion for 90 minutes), one in an acute phase (3-4 post MI), one in a subacute phase (10-14 days post MI) and a last one in a chronic phase (55-64 days post MI). As part of the last scan, animals are euthanized after the *in vivo* MR protocol and a short *ex vivo* protocol is run *in situ*.

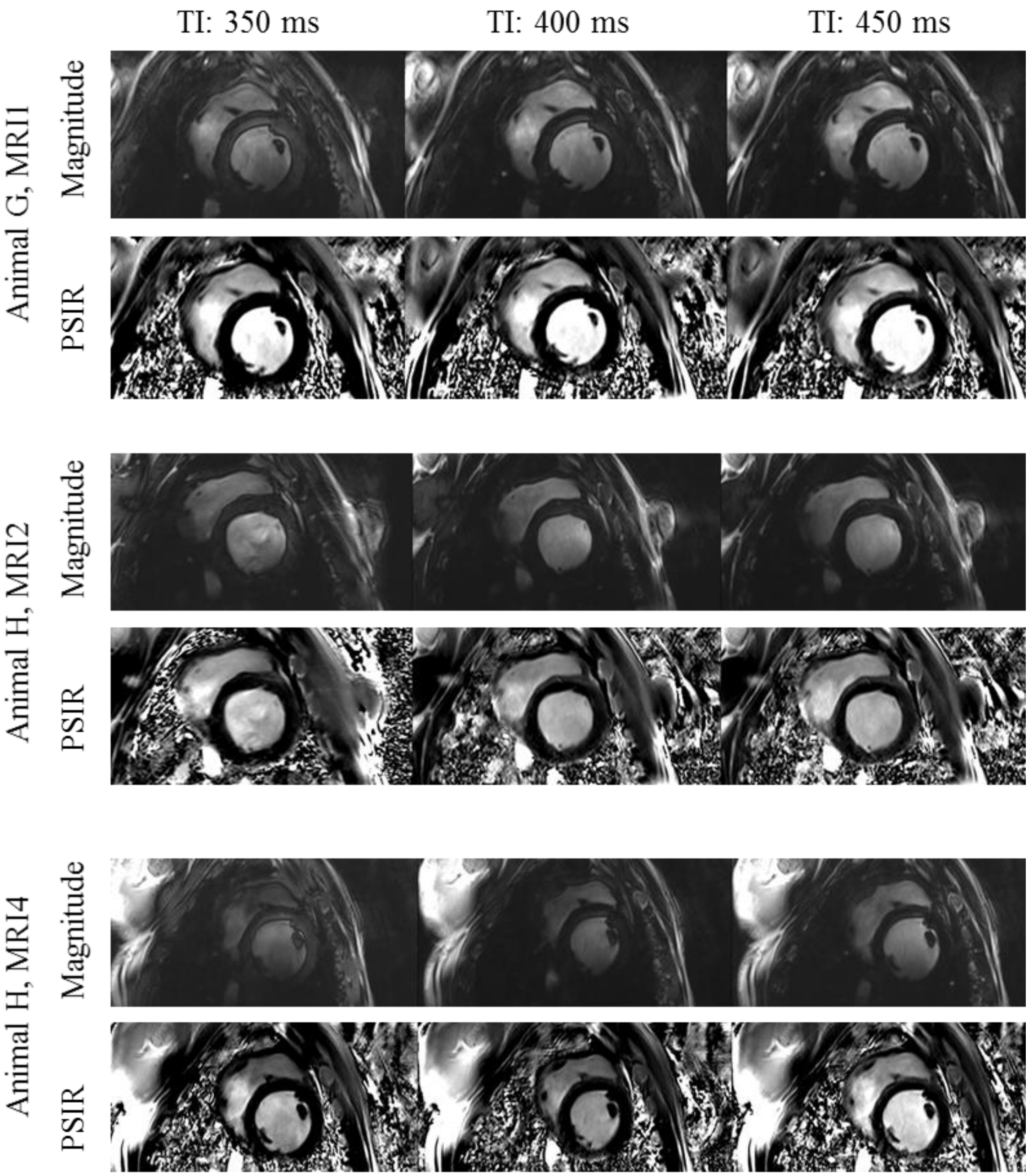

Supplemental Figure 3: Representative magnitude and PSIR LGE images acquired as TI scouts in this study using inversion times of 350, 400, and 450ms. 350 ms were found to be too short for magnitude images. 400 ms typically started to be adequate for nulled myocardium and was the inversion time of choice in some scans but showed lower blood tissue contrast compared to images acquired using an inversion time of 450 ms in other scans.

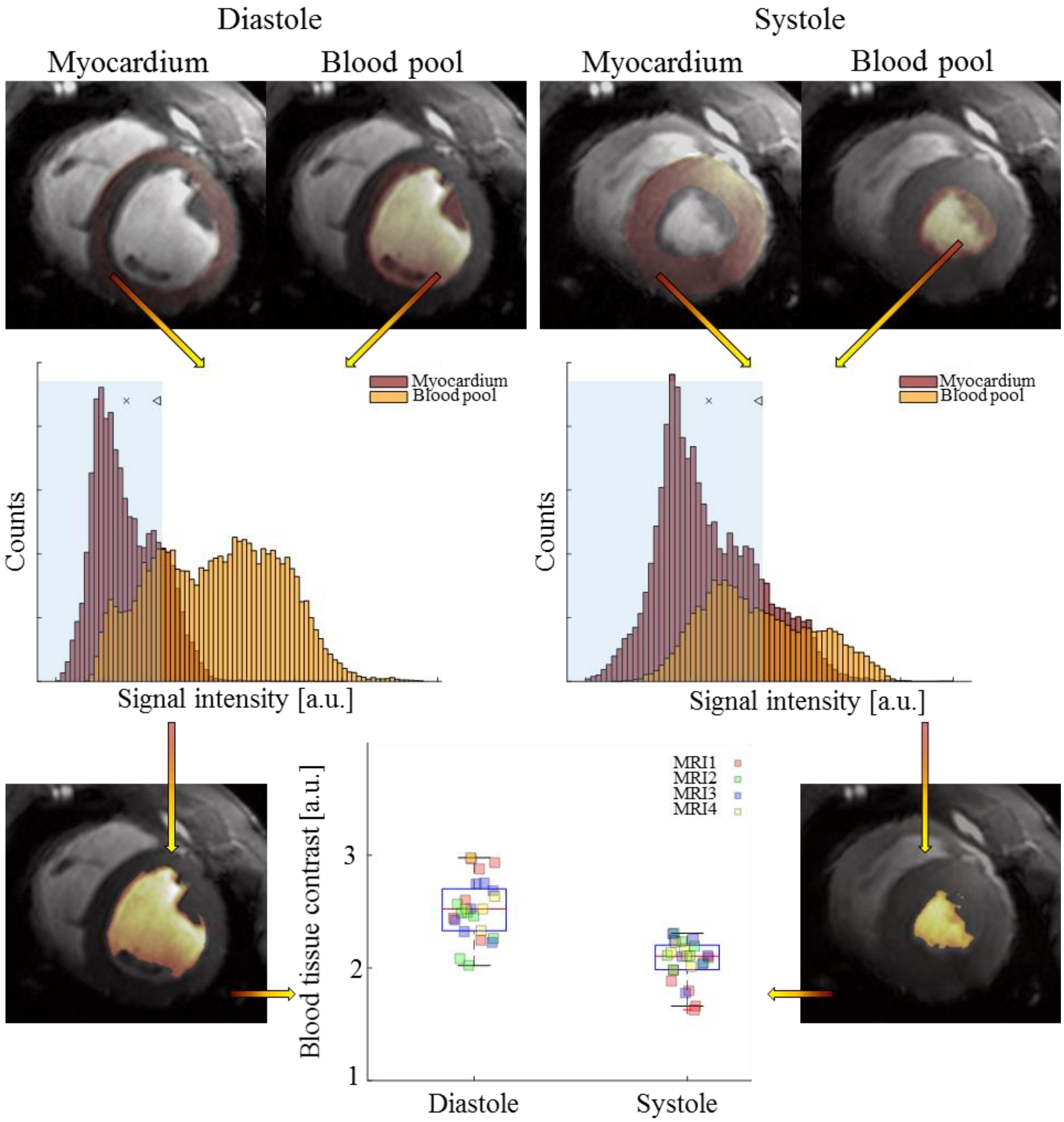

Supplemental Figure 4: Pipeline for the assessment of blood tissue contrast in systole and diastole. Representative segmentation of cine data for a basal slice in diastole and systole is shown in the top. Contours were exported from Medis and imported into Matlab. The area with red overlay corresponds to the myocardium, while the yellow area corresponds to the blood pool. Histograms show respective signal distributions. The blood pool segmentation exported from Medis includes papillary muscle and cannot directly be used to get an average blood pool signal. We therefore apply a filter that removes all voxels within a signal intensity of one standard deviation of the mean myocardial signal. An x in the histograms indicates the mean myocardial signal and the distance between the x and the triangle corresponds to one standard deviation. The blue background sums up all the values that are filtered from the blood pool signal. The displayed blood pool contours are post application of the filter. Papillary muscles are now excluded from the segmentation. The plot shows blood tissue contrast values for all measurements in diastole and systole.

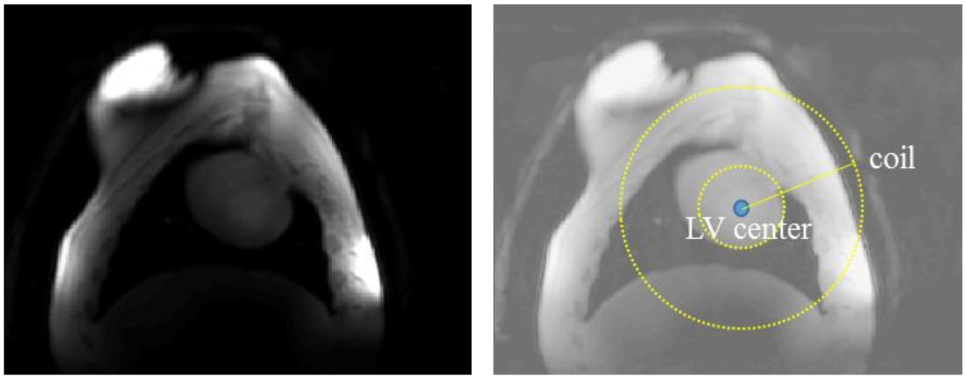

Original image, GRE (TE: 1.1ms) $\quad$ Log_{10}(image)

Supplemental Figure 5: Distance measurement from LV center to the coil housing. We used a mid-cavity slice with a short echo time from T_2* acquisitions. The very short echo time (TE: 1.1 ms) in combination with a logarithmic scale enabled visualization of the coil housing. In order to determine to LV center we created a circular ROI that was fit to epicardial borders of the LV. The center of gravity of this circle was determined as the center of the LV. A second circular ROI was drawn around the LV center. The radius of this circle was increased, until its boundaries met the coil. The shortest distance from LV center was then set as the distance between the LV center and the connection point of the outer circle with the coil housing.

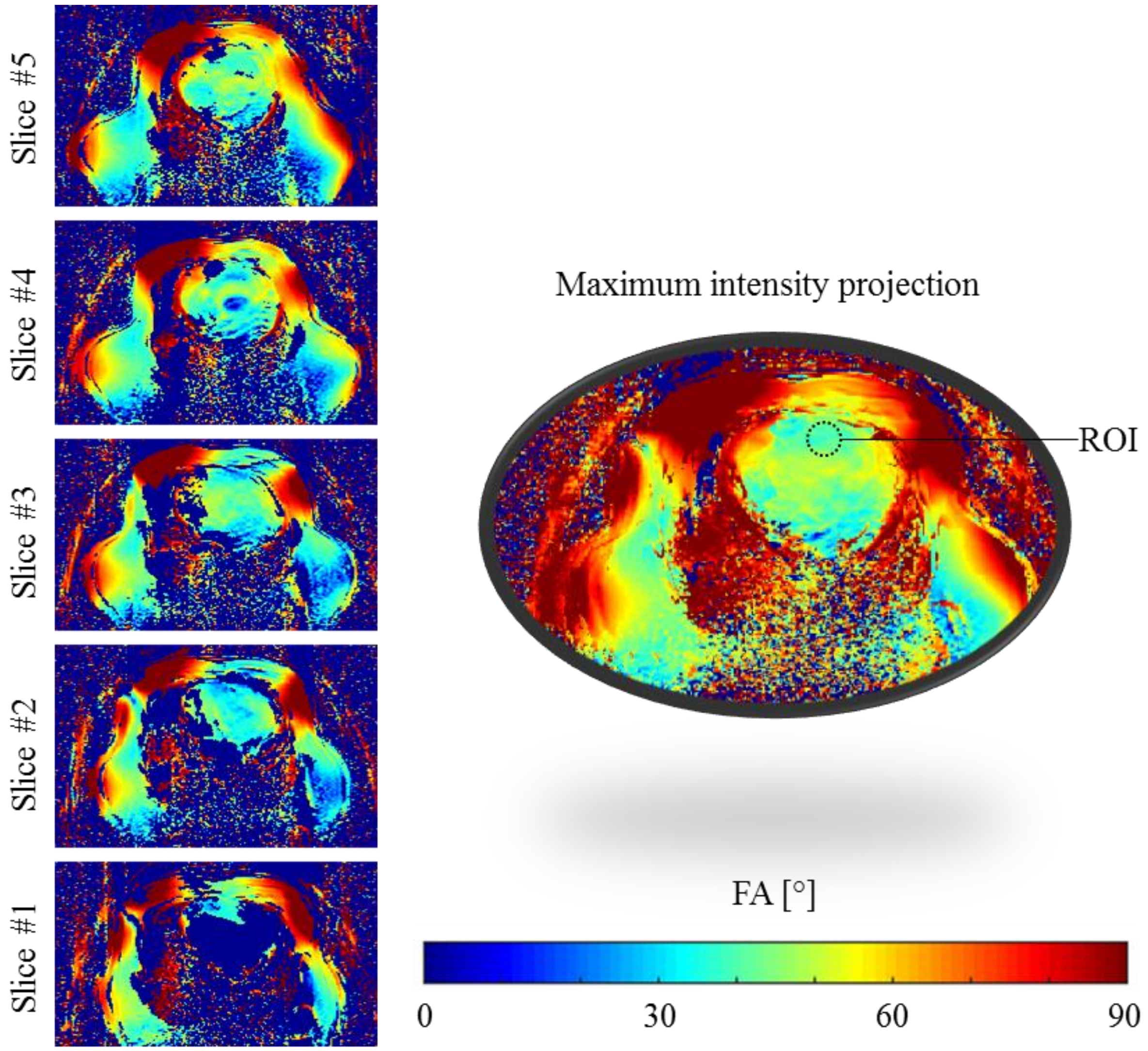

Supplemental Figure 6: Measurement of the flip angle for the B_1-normalization of SNR in acquired CINE images. The five central slices of the short axis stack of B_1 maps in transversal orientation are used to generate maximum intensity projections for each session. This process removes signal dropouts in the vendor reconstruction of individual B_1 maps. In these maximum intensity projections, a circular ROI is placed on the intersection of left and right ventricle. The average flip angle of this ROI is used in the normalization of derived SNR.

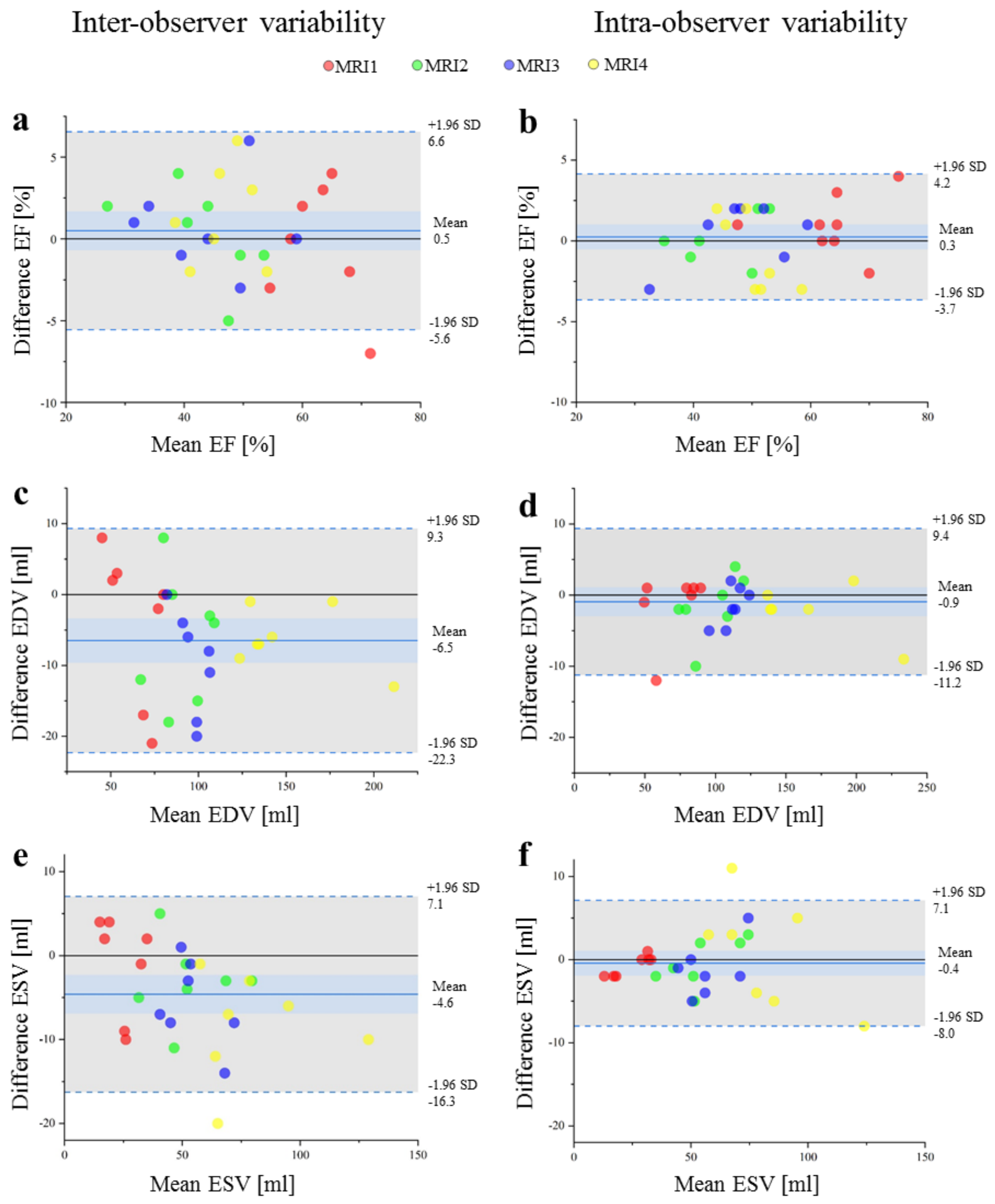

Supplemental Figure 7: Assessment of cardiac function in a large animal model of acute and chronic infarction. Bland-Altman plots for inter- and intra-observer variability in the metrics of EF, EDV and ESV. Gray shading depicts intervals of Mean ± 1.96 standard deviations. Color-coding in corresponds to measurement time points prior to and post MI. MRI indices refer to baseline (MRI1), acute (MRI2, 4±1 days post MI), sub-acute (MRI3, 12±1 days post MI), and chronic (MRI4, 58±2 days post MI) phases of the study.

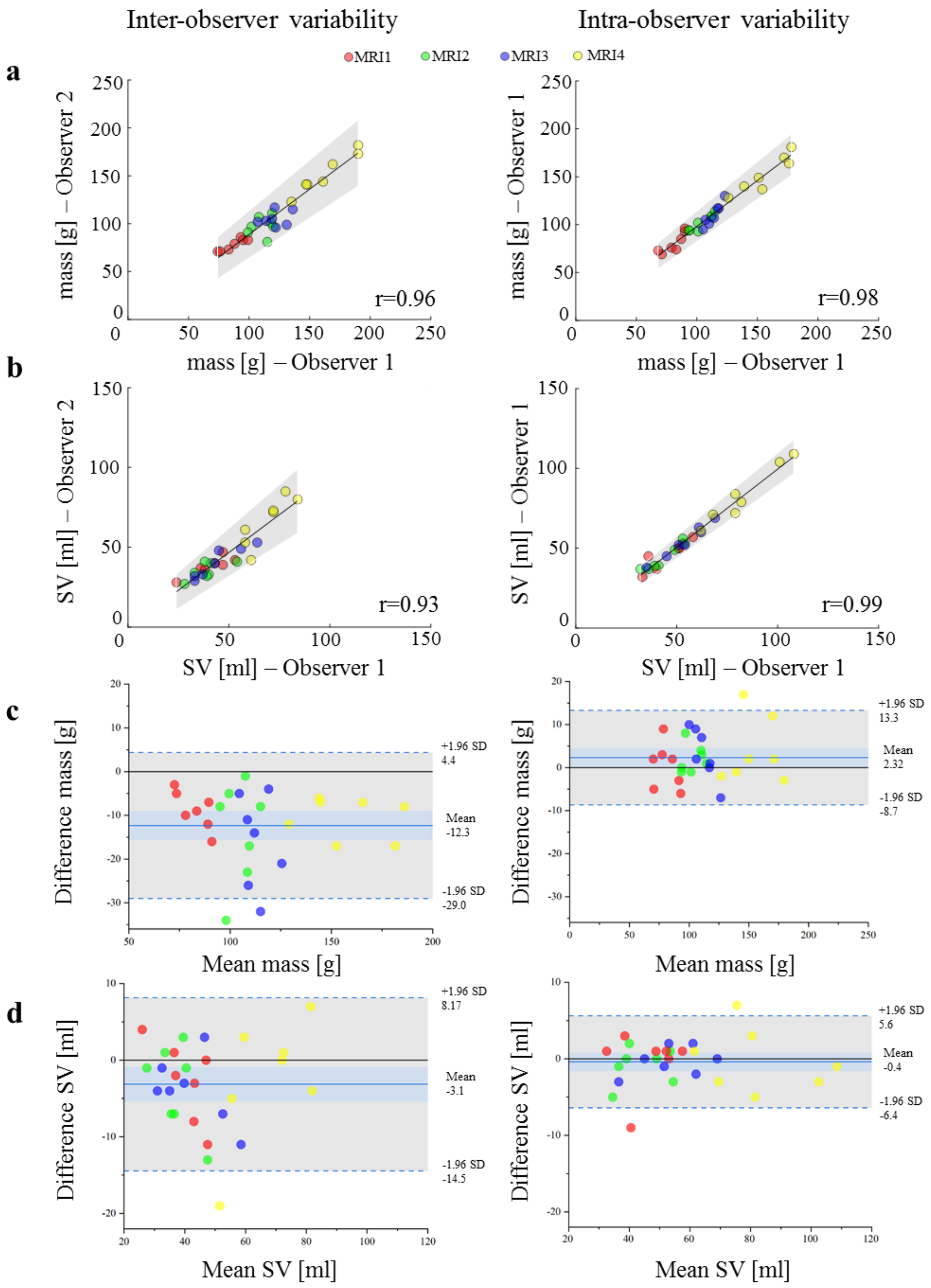

Supplemental Figure 8: Assessment of myocardial mass and stroke volume in a large animal model of acute and chronic infarction. a,b: Inter- and intra-observer correlation of myocardial mass and stroke volume. Gray shading in **a&b** indicates 95% confidence intervals of the fit. **c,d:** Bland-Altman plots for inter- and intra-observer variability of myocardial mass and stroke volume. Gray shading in **c&d** depicts intervals of Mean ± 1.96 standard deviations. Color-coding in all plots corresponds to measurement time points prior to and post MI. MRI indices refer to baseline (MRI1), acute (MRI2, 4±1 days post MI), sub-acute (MRI3, 12±1 days post MI), and chronic (MRI4, 58±2 days post MI) phases of the study.

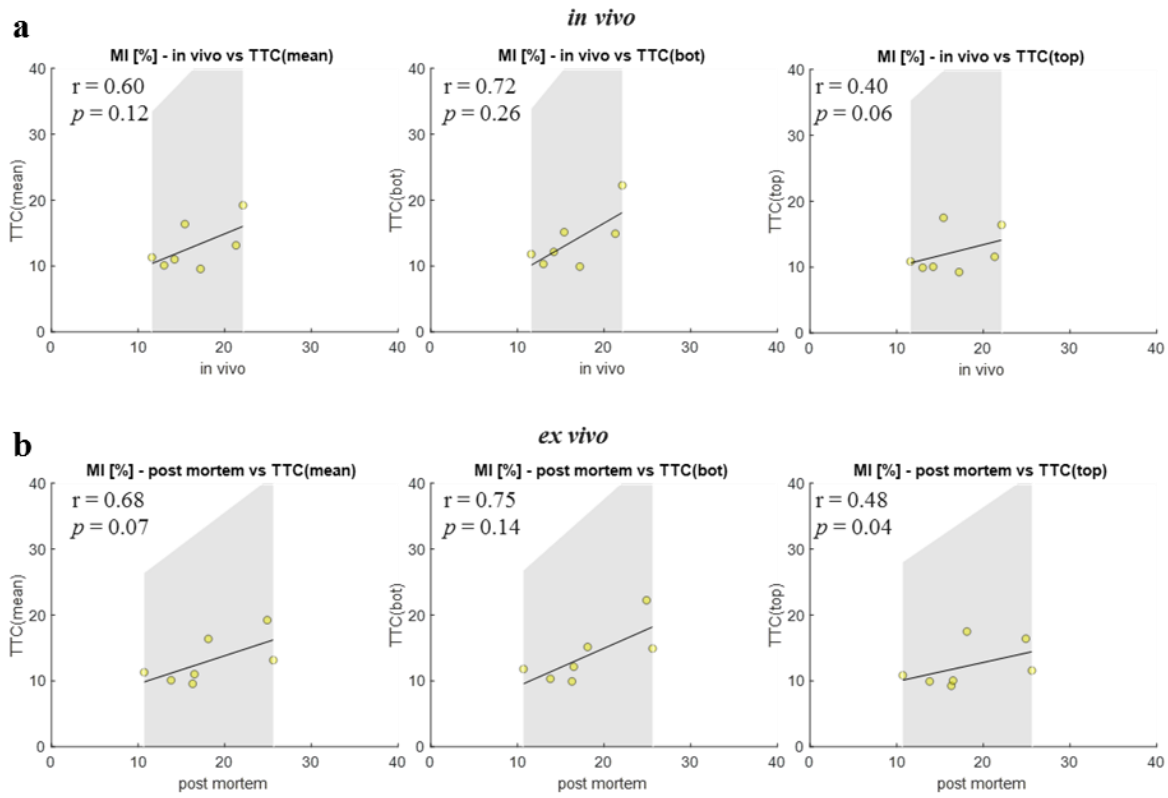

Supplemental Figure 9: Correlation between LGE data based on PSIR images and infarct sizes derived from different analysis approaches of TTC staining. a: Infarct sizes derived from TTC images compared to *in vivo* LGE. TTC images of every myocardial slice were acquired from the bottom (line of sight from apex to base) and the top (line of sight from base to apex). TTC(mean) denotes infarct sizes determined as the mean of bottom and top view results, TTC(bot) from the bottom view, and TTC(top) from the top view. **b:** Infarct sizes derived from TTC images compared to *ex vivo* LGE. Gray shading indicates 95% confidence intervals of the fit. Values for statistical significance are based on a paired t-test.

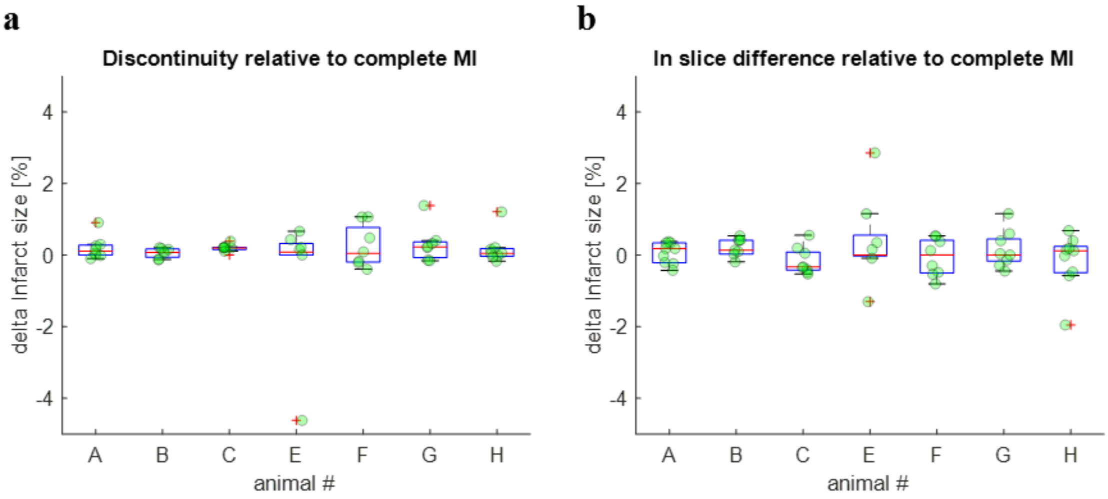

Supplemental Figure 10: Method dependent influences on infarct sizes derived from TTC data. a: Impact of slice discontinuity on infarct size quantification relative to total infarct volume for all animals. Discontinuity here means the difference in infarct size determined for the bottom and top view of two adjacent slices, which in theory, should be equivalent. Differences may be the result of sawing for tissue processing or just related to segmentation. **a:** Impact of the line of sight on infarct size quantification relative to total infarct volume for all animals. Line of sight here means the difference in infarct size determined for the bottom (line of sight from apex to base) and the top (line of sight from base to apex) view of the same slice.

Supplemental Video

Supplemental Video 1: UHF cardiac CINE images in a large animal with acute and chronic infarction. Representative basal, mid-cavity, and apical CINE images of the same animal (G) prior to and at three time points after myocardial infarction. Wall motion abnormalities are visible in the apical slices post myocardial infarction. Susceptibility effects are present in the infarct region 3 days post MI and remain at the tissue-blood-boundary in the sub-acute and chronic stage. Top: Complete field of view, illustrating the extend of a 60-day growth period for German Landrace pigs. Bottom: cropped section of the heart, showing the consistency in blood tissue contrast.

Supplemental Video 2: LGE short axis stack measured both in vivo and in situ 57 days post MI. For *in vivo* data, PSIR images show infarcted areas more clearly compared to in situ scans. For PSIR data, artificially high signal intensities are visible in the lateral wall for both *in vivo* and *in situ* acquisitions.

References

1. Selvakumar D, Deshmukh T, Foster SL, et al. Comparative assessment of motion averaged free-breathing or breath-held cardiac magnetic resonance imaging protocols in a porcine myocardial infarction model. *Sci Rep.* 2022;12(1):3727.
