# Supplementary material for: Precision imaging of cardiac function and scar size in acute and chronic porcine myocardial infarction using ultrahigh-field MRI
Source: Commun Med (Lond). 2024 Jul 18;4:146. doi: 10.1038/s43856-024-00559-y (PMC11258271; doi:10.1038/s43856-024-00559-y)
Supplement: Supplementary file 2 — Description of Additional Supplementary Files [file 43856_2024_559_MOESM2_ESM.pdf]

Description of Additional Supplementary Files

File name: Supplemental Video 1

File Description: UHF cardiac CINE images in a large animal with acute and chronic infarction. Representative basal, mid-cavity, and apical CINE images of the same animal (G) prior to and at three time points after myocardial infarction. Wall motion abnormalities are visible in the apical slices post myocardial infarction. Susceptibility effects are present in the infarct region 3 days post MI and remain at the tissue-blood-boundary in the sub-acute and chronic stage. Top: Complete field of view, illustrating the extend of a 60 day growth period for German Landrace pigs. Bottom: cropped section of the heart, showing the consistency in blood tissue contrast.

File name: Supplemental Video 2

File Description: LGE short axis stack measured both *in vivo* and in *situ* 57 days post MI. For *in vivo* data, PSIR images show infarcted areas more clearly compared to *in situ* scans. For PSIR data, artificially high signal intensities are visible in the lateral wall for both *in vivo* and *in situ* acquisitions.
